# Programmed cell death activated by Rose Bengal in *Arabidopsis thaliana* cell suspension cultures requires functional chloroplasts

**DOI:** 10.1093/jxb/eru151

**Published:** 2014-04-10

**Authors:** Jorge Gutiérrez, Sergio González-Pérez, Francisco García-García, Cara T. Daly, Óscar Lorenzo, José L. Revuelta, Paul F. McCabe, Juan B. Arellano

**Affiliations:** ^1^Instituto de Recursos Naturales y Agrobiología de Salamanca (IRNASA-CSIC), Cordel de merinas 52, 37008 Salamanca, Spain; ^2^Department of Nutritional Sciences, School of Biosciences and Medicine, Faculty of Health and Medical Sciences, University of Surrey, Guildford, Surrey GU2 7XH, UK; ^3^Functional Genomics Node, INB, Computational Medicine, Prince Felipe Research Centre, Av. Autopista del Saler 16, Camino de las Moreras, 46012 Valencia, Spain; ^4^School of Science, Department of Chemical and Life Sciences, Waterford Institute of Technology, Cork Road, Waterford, Ireland; ^5^School of Biology and Environmental Science, University College Dublin, Belfield, Dublin 4, Ireland; ^6^Departamento de Fisiología Vegetal, Centro Hispano-Luso de Investigaciones Agrarias, Facultad de Biología, Universidad de Salamanca, C/ Río Duero 12, 37185 Salamanca, Spain; ^7^Departamento de Microbiología y Genética, Universidad de Salamanca, Campus Miguel de Unamuno, E-37007 Salamanca, Spain

**Keywords:** *Arabidopsis* cell cultures, photosensitizers, programmed cell death, reactive oxygen species, Rose Bengal, singlet oxygen, transcriptional defence responses.

## Abstract

Light-grown *Arabidopsis thaliana* cell suspension culture (ACSC) were subjected to mild photooxidative damage with Rose Bengal (RB) with the aim of gaining a better understanding of singlet oxygen-mediated defence responses in plants. Additionally, ACSC were treated with H_2_O_2_ at concentrations that induced comparable levels of protein oxidation damage. Under low to medium light conditions, both RB and H_2_O_2_ treatments activated transcriptional defence responses and inhibited photosynthetic activity, but they differed in that programmed cell death (PCD) was only observed in cells treated with RB. When dark-grown ACSC were subjected to RB in the light, PCD was suppressed, indicating that the singlet oxygen-mediated signalling pathway in ACSC requires functional chloroplasts. Analysis of up-regulated transcripts in light-grown ACSC, treated with RB in the light, showed that both singlet oxygen-responsive transcripts and transcripts with a key role in hormone-activated PCD (i.e. ethylene and jasmonic acid) were present. A co-regulation analysis proved that ACSC treated with RB exhibited higher correlation with the conditional fluorescence (*flu*) mutant than with other singlet oxygen-producing mutants or wild-type plants subjected to high light. However, there was no evidence for the up-regulation of *EDS1*, suggesting that activation of PCD was not associated with the EXECUTER- and EDS1-dependent signalling pathway described in the *flu* mutant. Indigo Carmine and Methylene Violet, two photosensitizers unable to enter chloroplasts, did not activate transcriptional defence responses in ACSC; however, whether this was due to their location or to their inherently low singlet oxygen quantum efficiencies was not determined.

## Summary

*Arabidopsis* cell suspension cultures are used as a biological tool to better understand the key role of functional chloroplasts in singlet oxygen-mediated programmed cell death in plants.

## Introduction

Singlet oxygen (^1^O_2_) is a reactive oxygen species (ROS) that is formed constitutively in photosystem II (PSII) of plant chloroplasts. Plants can cope with the basal production of ^1^O_2_ under normal environmental conditions, but high levels of ^1^O_2_ are produced in response to excess excitation energy in PSII when photosynthetic activity is inhibited by stress or inhibitors (Mullineaux and Baker, 2010). Overproduction of ^1^O_2_ causes damage to lipids and proteins in the neighbourhood of PSII, leading to a decrease in photosynthetic efficiency and an inhibition of plant growth. Damage to the β-carotene molecules of the PSII reaction centre (RC) by ^1^O_2_ has also been reported and, interestingly, some of the β-carotene oxidation products have been proposed to be stress signals that mediate gene responses to ^1^O_2_ (Ramel *et al.*, 2012*a*, *b*). The dual role of ^1^O_2_ as a cytotoxic molecule and a signal molecule is now well established (Kim *et al.*, 2008; Triantaphylidès and Havaux, 2009; Fischer *et al.*, 2013) and much of what is known about the role of ^1^O_2_ as a signalling molecule comes from the conditional *fluorescence* (*flu*) mutant of *Arabidopsis* (op den Camp *et al.*, 2003; Danon *et al.*, 2005; Laloi *et al.*, 2007; Lee *et al.*, 2007). The *flu* mutant contains a mutation in a negative regulator of chlorophyll (Chl) biosynthesis that results in enhanced production of protochlorophyllide (Pchlide)—a potent, natural ^1^O_2_ photosensitizer—(Meskauskiene *et al.*, 2001; Kauss *et al.*, 2012). When seedlings of the *flu* mutant are dark adapted, Pchlide accumulates in thylakoids. After a dark to light shift, a surge in ^1^O_2_ production occurs in chloroplasts, eventually leading to cell death. Direct photodamage by ^1^O_2_ takes place in chloroplasts of the *flu* mutant, but the ongoing events responsible for the cell death are genetically mediated by two plastid proteins EXECUTER1 (EX1) and EX2 (Lee *et al.*, 2007; Kim *et al.*, 2008; Kim and Apel, 2013). In other words, programmed cell death (PCD) is the default defence response that becomes active in the *flu* mutant. The role of the EX proteins has also been extended to wild-type plants, but the defence responses triggered seem different. Kim and co-workers (2012) proposed that EX-dependent signalling induces the formation of microlesions, but not the disintegration of chloroplasts, a finding that was interpreted as an acclimation response that enhances stress resistance in wild-type plants. Another *Arabidopsis* mutant where the ^1^O_2_-mediated transcriptional responses have been investigated in detail is the double mutant *npq1lut2* that lacks zeaxanthin and lutein (Alboresi *et al.*, 2011). In *npq1lut2*, the accumulation of ^1^O_2_ is selectively enhanced; however, the transcriptional response does not initiate PCD, but rather the induction of genes whose function is to protect chloroplasts against the damaging effect of ROS. In two other mutants, where ^1^O_2_ production is selectively enhanced, *vte1 npq1*, a double mutant deficient in α-tocopherol and zeaxanthin, and *ch1*, a mutant deficient in Chl *b*, high light (HL) irradiance led to a large increase of ^1^O_2_ that induced cell death as a consequence of a direct ^1^O_2_ cytotoxic effect (Triantaphylidès *et al.*, 2008), although it has recently been recognized that *ch1* can acclimate to ^1^O_2_ if exposed to mild light stress first (Ramel *et al.*, 2013). ^1^O_2_-mediated transcriptional responses at HL irradiance were also studied in wild-type plants and cell suspension cultures of *Arabidopsis* (González-Pérez *et al.*, 2011; Gutiérrez *et al.*, 2011; Ramel *et al.*, 2012*b*). HL stress revealed a set of transcripts that overlapped significantly with those listed in the *flu* mutant after the dark to light shift; however, in contrast to the *flu* mutant, an acclimatory response responsible for an increased tolerance against a more severe photooxidative stress was activated instead of PCD. It is worth noting that in wild-type plants ^1^O_2_ is produced at the heart of PSII (i.e. the PSII RC) and that the above studies provided evidence for ^1^O_2_ production in the PSII RC, for example non-enzymatic oxidation of the β-carotene of the PSII RC (Arellano *et al.*, 2011; Ramel *et al.*, 2012*a*) and photodamage to the D1 protein of the PSII RC (González-Pérez *et al.*, 2011). Furthermore, Ramel and co-workers (2012*b*) established that the differential transcriptional expression induced by β-carotene oxidation products was not mediated by the EX1 protein, and González-Pérez and co-workers (2011) showed no evidence for the up-regulation of *EDS1* encoding the ENHANCED DISEASE SUSCEPTIBILITY PROTEIN 1, also a gene key for understanding the ^1^O_2_-mediated cell death response (Ochsenbein *et al.*, 2006). At present, it is not well established whether differences in the type of ^1^O_2_-mediated transcriptional defence responses depend on the source of ^1^O_2_ within the chloroplasts (i.e. PSII RC, thylakoid-bound Chl precursors, infiltrated artificial photosensitizers, etc.), the intensity of the stress (time, concentration, etc.), or a combination of both, and questions about this issue have been raised in the past few years (Galvez-Valdivieso and Mullineaux, 2010; Alboresi *et al.*, 2011; Gutiérrez *et al.*, 2011; Ramel *et al.*, 2012a; Kim and Apel, 2013).

In an attempt to shed more light on the ^1^O_2_-mediated cell death response in plants, Rose Bengal (RB)—a potent, artifical ^1^O_2_ photosensitizer—and dark-grown and light-grown *Arabidopsis* cells, the former containing proplastids, while the latter have functional chloroplasts, were used here. RB is an ^1^O_2_ elicitor that accumulates inside chloroplasts and has been used in several studies with the model alga *Chlamydomonas reinhardtii* to investigate the role of ^1^O_2_ in chloroplast to nucleus retrograde signalling and acclimatory responses to ^1^O_2_ stress (Leisinger *et al.*, 2001; Fischer *et al.*, 2007; Ledford *et al.*, 2007). In these studies, when cells were exposed to light, RB induced (in a similar fashion to HL stress) the transcription of *GLUTATHIONE PEROXIDASE HOMOLOGOUS* (*GPXH*), a gene with a key role in defending against photooxidative stress. In addition, constitutive overexpression of *GPXH* along with *GLUTATHIONE-S-TRANSFERASE 1* (*GSTS1*) was sufficient to augment ^1^O_2_ resistance. As with *C*. *reinhardtii*, *Arabidopsis* cell suspension cultures (ACSC) are an excellent plant model system to investigate ^1^O_2_ elicitor-mediated transcriptional defence responses and cell viability (or PCD) following chemical treatments. Quantification of PCD rates in cells *in vivo* can be rather difficult when infiltration of chemicals is required as the defence responses often occur in a small group of inaccessible cells buried in a bulk of surrounding healthy cells. Consequently, plant cell cultures can offer a more suitable means of investigating PCD due to their accessibility, reduced complexity, and uniformity (McCabe and Leaver, 2000). Additionally, ACSC can also be grown in the dark without difficulty, which in turn represents another advantage when chloroplast-dependent physiological responses are under study (Doyle *et al.*, 2010).

In this study, ACSC were subjected to mild photooxidative conditions by adding RB at submicromolar concentrations to avoid side effects that were not directly related to ^1^O_2_ production. After the treatment with RB in the light, ^1^O_2_-mediated PCD was only observed in *Arabidopsis* cells containing functional chloroplasts, and their transcriptional defence responses resembled those induced by ^1^O_2_ in the *flu* mutant. The comparative analysis of the transcriptional profiles of ACSC, treated with RB and H_2_O_2_ in the light, provided some clues about transcripts with a key role in PCD. In order to test if cellular location was an important factor in responses induced by ^1^O_2_, two other ^1^O_2_ elicitors were used in this study, Methylene Violet (MV) and Indigo Carmine (IC), which accumulate differently in plant cells (Hideg *et al.*, 2011). However, neither was able to induce any statistically significant change in transcript expression. The poor ^1^O_2_ quantum yield of MV and IC in comparison with that of RB (Kovács *et al.*, 2013) and, consequently, the need for higher concentrations to match equal levels of ^1^O_2_ production means that it cannot be established whether lack of change in transcript expression was due to different cellular location or possible limitations in their suitability as photosensitizers in studies where the matter of interest is the effect of ^1^O_2_ production by them, but not their own chemical toxicity.

## Materials and methods

### Growth conditions for *Arabidopsis* cell suspension cultures

Light-grown *Arabidopsis thaliana* L. (Columbia ecotype) cell suspension cultures (ACSC) were maintained in 200ml of liquid growth medium (Jouanneau and Péaud-Lenoël, 1967; Axelos *et al.*, 1992) as previously described (González-Pérez *et al.*, 2011). For comparative purposes, *Arabidopsis* cells were also grown in the dark when cell viability was under investigation (see below).

### Cellular location of ^1^O_2_ photosensitizers

Before proceeding with the photooxidative treatments, the location of RB, MV, and IC inside the cells of *Arabidopsis* was investigated by confocal microscopy. The absorption and emission spectral features of these three ^1^O_2_ photosensitizers have been previously described (Morrison *et al.*, 1997; Roberts *et al.*, 1998; Chang *et al.*, 2008). A 1ml aliquot of ACSC, previously treated with 1mM of each photosensitizer for 10min in the dark, was centrifuged at 120 *g* for 3min at 4 °C, washed three times with 2ml of 2.7mM KCl, 147.3mM NaCl, and 10mM sodium phosphate [phosphate-buffered salin (PBS)] pH 7.4, and finally resuspended in 1ml of 10mM PBS pH 7.4. The fluorescence emission was then monitored with a confocal microscope (model DM IRB; Leica Microsystems) following excitation of the three photosensitizers and Chl at 543nm and 633nm with an argon laser and a Triplet Dichroic 488/543/633 excitation beam splitter. The fluorescence emission of the selected photosensitizers and chloroplast Chl-binding proteins was collected at 560–620nm and at 680–700nm, respectively.

### Photooxidative treatments

Nine-day-old cultures with a cell density of ~125–150mg ml^–1^ fresh weight were split into two batches, acclimatized before the chemical treatment for 2h in the dark or continuous illumination at 150 μE m^–2^ s^–1^ at 24 ºC with gentle agitation, and then treated with the photosensitizers at 0.5 μM and 5 μM. Cultures were also treated with 0.5mM and 5mM H_2_O_2_ for comparative purposes. After 30min of chemical treatment, samples were collected, filtered, frozen in liquid nitrogen, and stored at –80 °C until further analysis. Light and dark treatments were carried out simultaneously, performed in duplicate, and replicated three times. Control cultures were also exposed to the same conditions either at 150 μE m^–2^ s^–1^ or in the dark.

### Photosynthetic activity

In order to determine the photosynthetic activity, the rate of oxygen production was measured polarographically using a Chlorolab 2 system (Hansatech Instruments, Norfolk, UK) at 20 °C. Samples containing equal amounts of fresh weight of cells were incubated in the dark for 1min before a 300 μE m^−2^ s^−1^ light-emitting diode source was switched on. The contribution of mitochondrial respiration to the rate of oxygen production was subtracted when the same experiment was repeated with a fresh sample in the dark. The photosynthetic oxygen evolution rate was expressed as μM min^–1^ g^–1^ of fresh weight of cells and the percentage of photosynthetic activity was referred to control cells. All the rate measurements were performed in triplicate.

### Protein oxidation analysis

The oxidative damage to the D1 protein (i.e. PsbA) of the PSII RC and general protein oxidation in ACSC were evaluated by western blot. Proteins were extracted from ~500mg of frozen cells as previously described (González-Pérez *et al.*, 2011). Protein samples were subjected to 6M urea, 12% (w/v) SDS–PAGE and transferred overnight to nitrocellulose membranes. Nitrocellulose membranes were stained with Ponceau S solution (Applichem, Darmstadt, Germany) for 1min to visualize the protein transfer and then destained with water. The nitrocellulose membranes were blocked with 1% (w/v) bovine seum albumin (BSA) in 0.6% (w/v) Tween-20, 150mM NaCl, 50mM TRIS-HCl, pH 7.4 (T-TBS). For the immunodetection of PsbA, the nitrocellulose membranes were incubated for 1h at 37 °C with polyclonal antibodies against the C-terminal region of PsbA (Agrisera, Vännäs, Sweden) using a dilution of 1:5000 in T-TBS. After extensive washing in T-TBS, the immunocomplexed membranes were probed for 1h at 37 °C with an anti-rabbit, peroxidase-linked secondary antibody using a dilution of 1:2000 in T-TBS, and were washed with T-TBS once again. The immunodetection of PsbA was visualized with the 3,3’-diaminobenzidine tetrahydrochloride (DAB) substrate kit (Pierce Biotechnology, Rockford, IL, USA) or with luminol (Bio-Rad Laboratories, Hercules, CA, USA). To detect general protein oxidation, the extracted proteins were chemically modified with 2,4-dinitrophenylhydrazine (DNPH) and the corresponding carbonylated proteins were identified following the steps described in the OxyBlot protein oxidation detection kit from Millipore (Millipore, Billerica, MA, USA), and visualized with luminol (Bio-Rad Laboratories).

### Indirect detection of ^1^O_2_

The production of ^1^O_2_ by RB was analysed in ACSC that were first broken in water or deuterium oxide (D_2_O), and then treated with RB at a concentration of 5 μM under continuous illumination at 150 μE m^–2^ s^–1^ or in the dark for 30min. After the treatment, the electrophoretic migration of the band of the D1/D2 heterodimer was inspected by western blot analysis, as described above, to evaluate ^1^O_2_ production qualitatively (Lupinkova and Komenda, 2004; Arellano *et al.*, 2011).

### Measuring cell death levels

After the (photo)oxidative treatments, dark-grown or light-grown cells were diluted 20 times into fresh culture medium and left in the dark for 24h to allow the progression of cell death-associated morphological changes. The vital stain fluorescein diacetate (FDA) was applied to ACSC to assess their cell viability. Cells were subsequently scored as alive, dead via necrosis, or dead via PCD using a UV-equipped light microscope according to the methods previously described (McCabe and Leaver, 2000; Reape *et al.*, 2008; Doyle *et al.*, 2010). Under UV radiation, a bright green fluorescence is observed in viable cells with an intact plasma membrane. Dead cells that displayed the hallmark PCD morphology consisting of a condensed protoplast, which had retracted away from the cell wall, were scored as dead via PCD. FDA-stained cells that did not exhibit bright green fluorescence or a retracted cell protoplast were scored as necrotic.

### Target transcripts to evaluate early ROS-mediated responses

Target transcripts to monitor the responses of ACSC under the assayed oxidative stress conditions were selected from lists containing ROS markers specifically up-regulated by ^1^O_2_ and H_2_O_2_ (op den Camp *et al.*, 2003; Gadjev *et al.*, 2006; Laloi *et al.*, 2007). The *DEFENSIN-LIKE FAMILY* (*DEF*) transcript named At2g43510 was chosen as a marker for general oxidative stress (Gadjev *et al.*, 2006), whereas the *PROFILIN FAMILY* (*PROF*) transcript named At2g19760 was selected as a housekeeping marker for internal reference (Laloi *et al.*, 2007). The primers designed to amplify the selected transcripts are given in Supplementary Table S1 available at *JXB* online.

### RNA isolation and RT–PCR analysis

RNA isolation and relative quantification of mRNA expression were performed as previously described (Livak and Schmittgen, 2001; Chomczynski and Sacchi, 2006; González-Pérez *et al.*, 2011). Other intermediate steps such as contaminating genomic DNA elimination, RNA reverse transcription, and reverse transcription–PCR (RT–PCR) runs are as previously described (González-Pérez *et al.*, 2011). Expression levels were normalized using the housekeeping gene *PROF* (At2g19760).

### Microarray experiments and data analysis

Transcriptional analyses were performed using Affymetrix GeneChip Arabidopsis genome ATH1 arrays (Affymetrix Inc., Santa Clara, CA, USA). The method to verify the quality of total, DNA-free RNA, and other technical details describing the microarray experiments are as previously described (González-Pérez *et al.*, 2011). Experiments were performed using three biological replicates. Principal components analysis (PCA) was used to explore the variability between the replicates (Johnson and Wichern, 1998). Data from microarrays were standardized using quantile normalization and the robust multi-array average (RMA) method (Bolstad *et al.*, 2003). Differential transcript expression was carried out using the Limma package (Smyth, 2004) available from Bioconductor (http://www.bioconductor.org/). Multiple testing adjustments of *P*-values were carried out according to the Benjamini and Hochberg methodology (Benjamini and Hochberg, 1995). A volcano plot was used to visualize transcripts with statistically significant differential expression (Cui and Churchill, 2003). Significantly over- or under-represented gene ontology (GO)/biological process terms were obtained using FatiScan from the Babelomics suite (http://babelomics.bioinfo.cipf.es/) as described previously (Al-Shahrour *et al.*, 2004, 2007; Medina *et al.*, 2010). Multiple testing adjustment of *P*-values was then carried out according to the false discovery rate (FDR) method (Benjamini and Hochberg, 1995; Benjamini and Yekutieli, 2001). GO terms were annotated from the Ensembl’s 56 (TAIR 10) release (http://www.ensembl.org).

### Validation of microarray experiments

In order to validate the microarray experiments, several transcripts with statistically significant differential expression were selected from the list included in Supplementary Tables S2 and S3 at *JXB* online. The selected transcripts and their corresponding primers are given in Supplementary Table S4.

### Co-regulation analysis

Data from the microarray experiments were clustered together with expression data from previously selected key treatments in *Arabidopsis* based on comparative analysis using Genevestigator (Hruz *et al.*, 2008). Only transcripts presenting signal Log_2_ ratios ≥1 (induction) or ≤ –1 (repression) were considered for analysis. The hierarchical clustering analysis was then carried out using R statistical software, version 2.12.1 (http://www.r-project.org). Pearson’s correlation analysis was performed to evaluate the linear relationship between experimental treatments (Quinn and Keough, 2002). The raw microarray data have been deposited in the GEO database under the accession number GSE43551.

## Results

### Cellular location of the ^1^O_2_ photosensitizers in ACSC

**Fig. 1.**
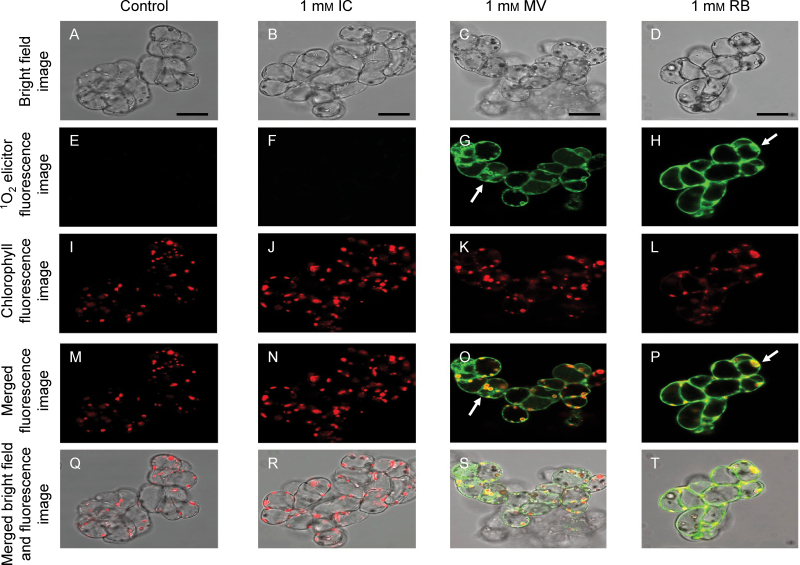
Representative confocal micrographs illustrating the location of IC, MV, and RB at a concentration of 1mM in ACSC. From top to bottom the following panels are represented: bright field images (A–D), ^1^O_2_ elicitor fluorescence images at 560–620nm (E–H), red Chl autofluorescence images at 680–700nm (I–L), merged Chl and ^1^O_2_ elicitor fluorescence images (M–P), and merged bright field and fluorescence images (Q–T). The ^1^O_2_ elicitor and red Chl fluorescence emissions were collected after excitation at 543nm and 633nm with an argon laser. Arrows in G, H, O, and P point to key features (see text for further details). Scale bars=50 μm.

The cellular location of the ^1^O_2_ photosensitizers RB, MV, and IC was investigated on the basis of their fluorescence using a confocal microscope (Fig. 1). Additionally, red Chl autofluorescence was used to identify the chloroplasts inside the cells (Fig. 1I–L). The incubation with each photosensitizer was performed in the dark for only 10min to avoid unnecessary cellular damage. The *Arabidopsis* cells treated with 1mM IC did not exhibit fluorescence emission (Fig. 1F), suggesting that this dye barely permeates the cell or it was not retained within the cell after washing with 10mM PBS pH 7.4. In contrast to IC, the *Arabidopsis* cells treated with 1mM MV or 1mM RB showed a distinct green fluorescence around and inside the cells that clearly indicated that these photosensitizers were both able to cross the plasma membrane and to be partly retained on the cell boundary (i.e. cell wall and plasma membrane), and other cellular compartments (Fig. 1G, H). In spite of the ability of MV and RB to cross the cell boundary, a closer inspection of the fluorescence images revealed a significant difference between the dyes. MV accumulated in the chloroplast envelope and showed a ring-like fluorescence emission around these organelles, while the red Chl autofluorescence was still discernible (Fig. 1G, O), indicating that MV apparently did not reach the chloroplast interior. In contrast, the distinctive red Chl autofluorescence was blurred by the green fluorescence emission of RB (Fig. 1H, P), showing clearly that this dye could indeed cross the chloroplast envelope and accumulate inside this organelle.

### Protein oxidation in ACSC as marker for photooxidative stress level

**Fig. 2.**
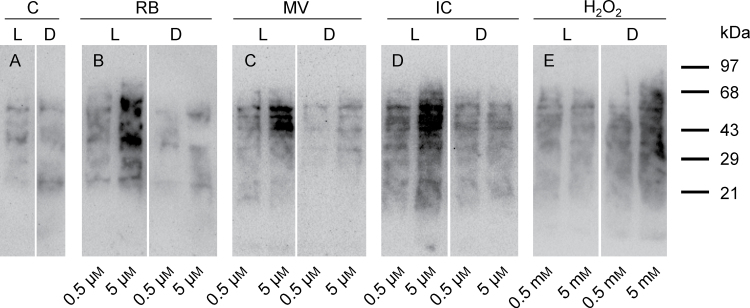
Protein oxidation analysis OxyBlot of ACSC under (photo)oxidative stress conditions. Control (A), RB (B), MV (C), IC (D), and H_2_O_2_ (E). ACSC were treated for 30min under continuous illumination at 150 μE m^−2^ s^−1^ (L) or in the dark (D) at two different concentrations for each photosensitizer (0.5 μM and 5 μM) and H_2_O_2_ (0.5mM and 5mM). Each lane was loaded with 30 μg of protein.

Experimental conditions were chosen to induce mild photooxidative stress in *Arabidopsis* cells in order to investigate defence responses that would be activated in a series of scenarios where the partitioning of ^1^O_2_ between the surrounding medium, the cells, and cellular organelles was imposed by the chemical nature of the ^1^O_2_ photosensitizers. Additionally, *Arabidopsis* cells were exposed to H_2_O_2_ for comparative purposes, and its concentration was adjusted to induce a level of oxidative stress similar to that caused by the three ^1^O_2_ elicitors. In order to avoid the superimposition of other signalling pathways that could become activated by severe damage, the lowest possible concentrations of RB, MV, IC, and H_2_O_2_ were chosen where oxidative damage was perceived in the first 30min of the light treatment. The level of carbonylated proteins was measured as a marker for oxidative stress in the cellular proteome of ACSC. Figure 2 shows the changes in the oxidation state of the cell proteome after the 30min treatment with two different concentrations for RB, MV, IC (i.e. 0.5 μM and 5 μM), and for H_2_O_2_ (i.e. 0.5mM and 5mM). A light treatment of ACSC with the photosensitizers at a concentration of 10 μM after 30min showed clear evidence of cellular lysis and a loss of cellular material after filtration, particularly in the case of RB. Additionally, homogenous light penetration in the culture medium was hampered at concentrations >10 μM due to the high molar absorptivity of the photosensitizers. Consequently, the concentration of 5 μM for each photosensitizer was considered as the upper limit for this photooxidative analysis. In the absence of the photosensitizers or H_2_O_2_, the OxyBlot experiments showed the immunodetection of a basal level of protein oxidation that did not differ appreciably between light and dark conditions after 30min (Fig. 2). This was not the case when the photosensitizers were added to the medium. The light treatments of ACSC in the presence of RB, MV, and IC at 5 μM revealed an evident increase in protein oxidation in their respective lanes that was not accompanied by similar changes in the dark treatments for the same concentration, showing a clear light-dependent effect on the protein oxidation of ACSC. The changes in protein oxidation of ACSC at 0.5 μM were subtle, but still discernible, for each photosensitizer when the respective treatments in light and dark conditions were compared. Therefore, 0.5 μM for each photosensitizer was considered high enough to induce mild photooxidative stress. When compared with control ACSC, the treatment of ACSC with H_2_O_2_ enhanced protein oxidation, but it was not light dependent. On the contrary, protein oxidation became more apparent in the dark, although this finding was not investigated further.

### RT–PCR analysis of selected transcripts responding to photooxidative stress

Changes in the expression profile for some specific markers for ^1^O_2_ and H_2_O_2_ were investigated after a 30min treatment with RB, MV, and IC at 0.5 μM, and H_2_O_2_ at 500 μM. For the sake of clarity, the full name of the chosen specific markers and their respective locus identifiers are given in Supplementary Table S1 at *JXB* online. As shown in Supplementary Table S5, significant changes were observed in the expression profile of some selected markers after the treatments with RB. However, no significant changes in the expression profile of any of the selected ROS markers were observed after the treatments with IC and MV, a result that was further confirmed in the whole-genome transcriptional profiling analysis (see below). When ACSC were exposed to RB, the specific ^1^O_2_ markers *NOD* and *SZF1* were significantly up-regulated, whereas the specific H_2_O_2_ marker *SUBT* was found to be down-regulated. The same ROS markers (i.e. *NOD*, *SZF1*, *SUBT*, and *F9D12*) did not show any significant change under dark conditions during the 30min treatment with 0.5 μM RB. No significant changes in the expression profile of the general oxidative stress marker (*DEF*) were observed in the presence of any of the ^1^O_2_ elicitors. In contrast to this, the 30min treatment with H_2_O_2_ resulted in slight up-regulation of *DEF*, but not of any of the selected H_2_O_2_ markers, confirming that 500 μM H_2_O_2_ also induced mild oxidative stress in ACSC under the experimental conditions used here.

### Limma analyses of ^1^O_2_- and H_2_O_2_-mediated transcriptional responses of ACSC

The ^1^O_2_- and H_2_O_2_-mediated transcriptional responses of ACSC were further characterized using Affymetrix GeneChip *Arabidopsis* genome ATH1 arrays. The microarray experiments were designed and classified as follows: control (C1, C2, and C3), RB at 0.5 μM (RB1, RB2, and RB3), MV at 0.5 μM (MV1, MV2, and MV3), IC at 0.5 μM (IC1, IC2, and IC3), and H_2_O_2_ at 500 μM (HP1, HP2, and HP3); where the numbers 1–3 represent the number of biological replicates. After data normalization, the Limma package identified a total of 1705 transcripts which were differentially expressed in ACSC exposed to RB with an adjusted *P-*value <0.05 (Supplementary Table S2 at *JXB* online). Out of a total 1705 transcripts, 314 had a fold change >1 (Log_2_), whereas 171 exhibited a fold change < –1. Surprisingly, no transcripts with statistically significant differential expression (adjusted *P*-value <0.05) were found in cell cultures treated with either MV or IC. In comparison with the RB treatment, the treatment of ACSC with 500 μM H_2_O_2_ induced a mild transcriptional response characterized by the observation that both the number of transcripts differentially expressed (i.e. 568, adjusted *P*-value <0.05) and the number of transcripts with a fold change < –1 or > 1 (i.e. 270) were lower than in the RB treatment (Supplementary Table S3). Volcano plots are shown in Supplementary Fig. S1 to better summarize the experimental conditions that yielded statistically significant changes in transcript expression. It is worth noting that 32 out of the 314 up-regulated transcripts (and four out of the 171 down-regulated transcripts) with a statistically significant fold change |Log_2_|>1 after treatment with 0.5 μM RB were included in the lists of 296 up-regulated and 29 down-regulated transcripts described as specific markers for ^1^O_2_ according to the transcriptional profile analyses performed by Gadjev *et al.*, (2006). Approximately 45% and 30% of the transcripts with up- and down-regulation after the treatment with 500 μM H_2_O_2_ were also present in the lists of up- and down-regulated transcripts after treatment with 0.5 μM RB. Surprisingly, the treatment with 500 μM H_2_O_2_ induced the expression (|Log_2_|>1) of only three transcripts defined as specific markers for H_2_O_2_, but 19 transcripts defined as specific markers for ^1^O_2_ (Gadjev *et al.*, 2006) (Supplementary Table S6).

RT–PCR and Limma analyses were in line with respect to the treatments with 0.5 μM MV and 0.5 μM IC, and both confirmed that there were no statistically significant changes in the transcriptional profile of ACSC after these treatments. It was first hypothesized that the different cellular location of the three photosensitizers could explain the divergent changes in the transcriptional profile. However, it was also found reasonable that the differences were based on the inherent quantum yields of ^1^O_2_ for RB, MV, and IC, which are in a ratio of 0.75:0.37:0.095 (Kovacs *et al.*, 2013). Although the most obvious thing to do was to adjust the MV and IC concentrations to reach ^1^O_2_ production levels similar to RB, several factors such as cellular lysis, non-homogeneous light penetration, and a direct cytotoxic effect by the photosensitizers discouraged the use of higher concentrations. Therefore, it could not be unambiguously determined whether cellular location was the sole reason why no significant changes in the transcriptional profile of ACSC were observed after the treatments with IC and MV, and, consequently, the study was centred on the treatments with RB and H_2_O_2_.

### Microarray data validation by RT–PCR

The microarray data of the 30min treatments with 0.5 μM RB or 500 μM H_2_O_2_ were confirmed by RT–PCR. All the transcripts selected for validation (Supplementary Table S4 at *JXB* online) showed similar fold changes in expression when they were analysed by both techniques (Supplementary Fig. S2).

### GO process terms associated with response to stimuli after (photo)oxidative treatments

In an attempt to identify GO/biological process terms that became over-represented after the treatments with RB and H_2_O_2_, functional enrichment was carried out using FatiScan from the Babelomics suite (http://babelomics.bioinfo.cipf.es). Briefly, this tool makes use of the complete list of genes available in the ATH1 arrays (i.e. ~24 000) and detects significantly up- or down-regulated blocks of functionally related transcripts ordered by differential expression after the treatment, no matter whether the differential transcriptional expression is found to be statistically significant or not. The treatment with 0.5 μM RB exhibited an extensive list of over-represented biological processes (42 GO terms) that were associated with responses to (i) stimuli (i.e. 25 out of 42; defence and immune responses, responses to abiotic, biotic, organic substance, etc.) and (ii) metabolic, cellular, and developmental processes (i.e. 17 out of 42; nitrogen, nucleic acid, and protein metabolic processes, transcription, protein folding and modification, cell cycle, PCD, apoptosis, etc.) (Supplementary Table S7 at *JXB* online). In the presence of 500 μM H_2_O_2_, 46 GO terms were over-represented with statistical significance and they could be divided into GO terms ascribed to responses to (i) stimuli (i.e. 20 out of 46) and (ii) metabolic, cellular, and developmental processes (i.e. 27 out of 46) (Supplementary Table S8). Sixteen out of the 20 over-represented defence processes for the treatment with H_2_O_2_ were common with the RB treatment (Supplementary Tables S7 and S8). Other GO/defence process terms for the H_2_O_2_ treatment were associated with metal ion responses.

### RB and H_2_O_2_ inhibit the photosynthetic activity of ACSC

**Fig. 3.**
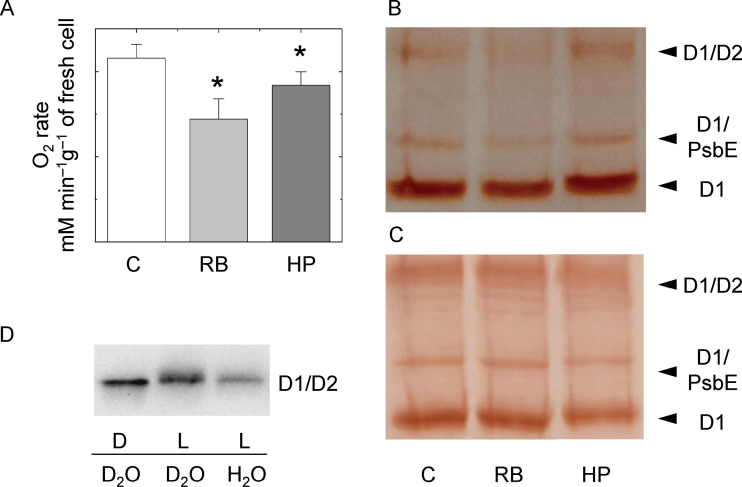
(A) Oxygen evolution rates of ACSC under control conditions (C) and after the 30min treatments with 0.5 μM RB and 500 μM H_2_O_2_, *P-value <0.05. (B and C) Western blot analysis of the D1 protein of PSII after the 30min treatment with 0.5 μM RB and 500 μM H_2_O_2_ (HP) under continuous illumination (B) and in the dark (C). Lane 1, control (C); lane 2, RB; lane 3, HP. Each lane was loaded with 70 μg of protein. The upper, middle, and lower band show the D1/D2 heterodimer of PSII, the adduct between the monomeric D1 protein and the α-subunit of the cytochrome *b*559 (D1/PsbE), and the monomeric D1 (D1), respectively. (D) Western blot analysis of the D2/D1 heterodimer of PSII after the 30min treatment of broken ACSC with 5 μM RB under continuous illumination at 150 μE m^−2^ s^−1^ (L) or in the dark (D) in water or deuterium oxide (D_2_O). Each lane was loaded with 30 μg of protein.

The photosynthetic oxygen evolution rate of ACSC was examined in the presence of 0.5 μM RB and 500 μM H_2_O_2_ (Fig. 3A). It decreased to ~65% of the initial value under control conditions when RB was present, suggesting that the ^1^O_2_ produced by RB inside chloroplasts could inhibit the photosynthetic activity of ACSC. The treatment with 500 μM H_2_O_2_ also brought a slight inhibition of the photosynthetic activity of ACSC, indicating that exogenous H_2_O_2_ easily permeated the plasma membrane and entered chloroplasts.

In order to establish whether the presence of ^1^O_2_ and H_2_O_2_ could damage the D1 protein of PSII in ACSC, cultured cells were subjected to a western blot analysis using an antibody raised against the D1 protein (Fig. 3B). In control conditions, three bands were distinguished; the lower and more intense band corresponded to the monomeric D1 protein of the PSII RC, whereas the upper band corresponded to the D1/D2 heterodimer of the PSII RC. The intermediate band, which is often observed to react against the D1 antibody, was a faint band that corresponded to an adduct between the D1 protein and the α-subunit of cytochrome *b*_559_ of PSII (Arellano *et al.*, 2011, and references therein). The intensity of the band ascribed to the monomeric D1 protein was lower in the presence of 0.5 μM RB, but it does not change in the treatment with 500 μM H_2_O_2_ (Fig. 3B). As a control experiment, the incubation with RB and H_2_O_2_ was also performed in the dark, and the results showed no damage to the D1 protein (Fig. 3C). Neither significant damage to the D1 protein nor a substantial decrease in the photosynthetic oxygen evolution rate were to be expected in the presence of 500 μM H_2_O_2_ on the basis of the study by Miyao *et al.* (1995), who reported a more visible and progressive damage to the D1 protein in the range of millimolar concentrations of H_2_O_2_. However, this does not exclude that other photosynthetic complexes (i.e. PSI or Rubisco) could be more sensitive to H_2_O_2_ inhibition (Nakano *et al.*, 2006).

To demonstrate qualitatively that RB produced ^1^O_2_ under the present experimental conditions, broken ACSC were treated with this photosensitizer at a concentration of 5 μM in both water and D_2_O, where the lifetime of singlet oxygen notably varies from ~4 μs to ~70 μs. It is known that the D1/D2 heterodimer exhibits a shift in its electrophoretic migration due to changes in protein conformation after oxidation of amino acid residues prone to react with ^1^O_2_ (Lupinkova and Komenda, 2004; Arellano *et al.*, 2011). Figure 3D showed that 5 μM RB induced a shift of the immunodetected band of the D1/D2 heterodimer toward the cathodic end of the gel together with a slight broadening after the light treatment. The shift was more prominent when the broken ACSC were exposed to the 30min light treatment in D_2_O, where the lifetime for ^1^O_2_ was longer and, consequently, the changes in the conformation of the D1/D2 heterodimer induced by ^1^O_2_ were more acute.

### RB, but not H_2_O_2_, affects PCD rates in light-grown ACSC

**Fig. 4.**
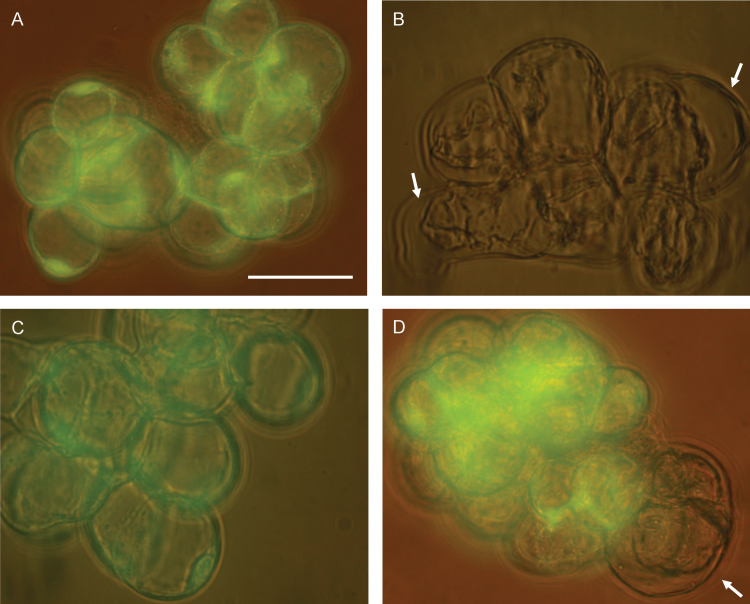
Representative field images showing changes in the morphology of light-grown *Arabidopsis* cells under control conditions (A), after the 30min treatment with 0.5 μM RB (B) and 500 μM H_2_O_2_ (C), and after the 90min treatment at 1800 μE m^−2^ s^−1^ (D). Bright green fluorescence of fluorescein diacetate following excitation with UV radiation in A, C, and D indicates that *Arabidopsis* cells remain viable after the stress treatment. Arrows in B and D illustrate some of the gaps between the cell wall and the plasma membrane after the induction of PCD. Scale bars=50 μm.

**Fig. 5.**
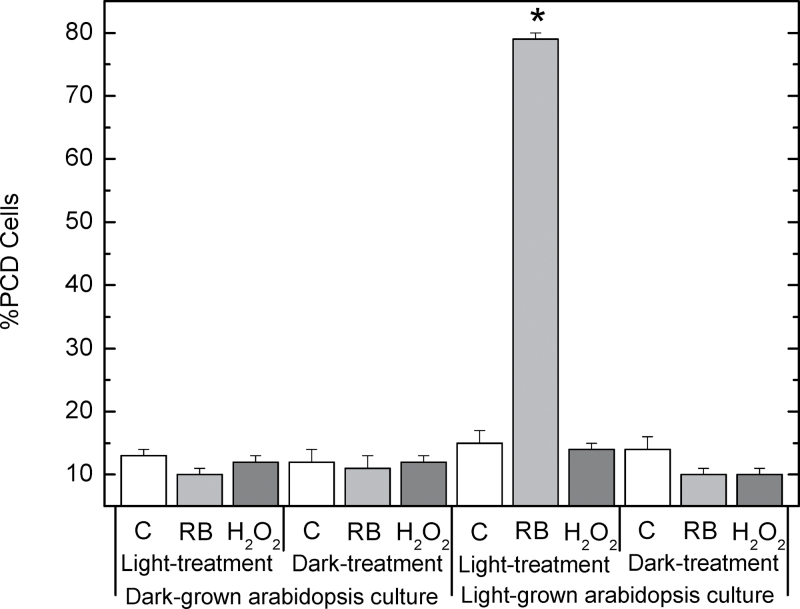
PCD in light-grown and dark-grown *Arabidopsis* cells subjected to 30min chemical treatments at 150 μE m^−2^ s^−1^ or in the dark. Black bar, 0.5 μM RB; grey bar, 500 μM H_2_O_2_; white bar, control samples. *P-value <0.05.

The production of ROS (and particularly ^1^O_2_) in chloroplasts has been demonstrated to play a key role in PCD regulation (Doyle *et al.*, 2010; Kim *et al.*, 2012). Therefore, the two treatments with RB or H_2_O_2_—both compounds able to enter chloroplasts—could affect the viability of ACSC and induce PCD. In order to determine if this was the case, visible morphological PCD changes, together with FDA fluorescence, were investigated. Figure 4 shows four representative bright field images of light-grown cells under control conditions, after treatment with 0.5 μM RB or 500 μM H_2_O_2_ for 30min under continuous illumination at 150 μE m^−2^ s^−1^, and after exposure to HL (i.e. 1800 μE m^−2^ s^−1^) for 90min. Under control conditions, light-grown cells exhibited the standard morphology (i.e. excessive hydration in cellular cultures) and were largely viable (12–15% PCD; Fig. 5), as demonstrated by the FDA fluorescence. In contrast to this, a clear gap between the cell wall and the plasma membrane was discernible in many of the light-grown cells treated with 0.5 μM RB, and bright green fluorescence could barely be detected in the cells stained with FDA (Fig. 4), indicating that PCD was greatly induced in light-grown ACSC after the RB treatment (79%; Fig. 5). The condensation of the cell protoplast could not be observed when light-grown cells were subjected to 500 μM H_2_O_2_, and bright green fluorescence was detected in the presence of FDA (Fig. 4). PCD was 14% (Fig. 5), indicating that the assayed concentration of H_2_O_2_ did not induce PCD significantly under these experimental conditions and that higher H_2_O_2_ concentrations are required for the activation of PCD (Desikan *et al*., 1998, 2001). Likewise, cell viability of light-grown cells exposed to HL was examined to explore whether ^1^O_2_ production by PSII was capable of activating PCD. HL treatment at 1800 μE m^−2^ s^−1^ for 30min did not affect cell viability (González-Pérez *et al.*, 2011) and only HL treatments for (or beyond) 90min resulted in morphological changes associated with PCD (Fig. 4). No significant effects were observed on PCD rates when light-grown ACSC were subjected to chemical treatments with 0.5 μM RB or 500 μM H_2_O_2_ for 30min in the dark (Fig. 5).

Further experiments were performed with dark-grown cells containing proplastids instead of functional chloroplasts (Doyle *et al.*, 2010), and cells were subjected to chemical treatments with 0.5 μM RB or 500 μM H_2_O_2_ for 30min under continuous illumination at 150 μE m^–2^ s^–1^ or in the dark. The results showed that the viability of the chemically treated cells was similar to that of control cells for dark-grown cells; there was no effect whether the chemical treatment was with 0.5 μM RB or 500 μM H_2_O_2_, or whether the cells were exposed to light or remained in the dark during the RB or H_2_O_2_ treatments (Fig. 5). Considered together, these results showed that the presence of developed chloroplasts and illumination were essential for the activation of PCD in ACSC treated with 0.5 μM RB.

### Co-regulation of ^1^O_2_-producing mutants and HL treatments with RB-treated ACSC

Over the last 14 years, analysis of the transcriptional profile of the *flu* mutant family has shed much light on ^1^O_2_-mediated defence responses in plants and has also provided lists of specific transcriptional markers and *cis*-regulatory elements (Meskauskiene *et al.*, 2001; op den Camp *et al.*, 2003; Gadjev *et al.*, 2006; Petrov *et al.*, 2012). The *flu* mutant family is characterized by the high production of ^1^O_2_ after the dark–light shift in response to the accumulation of Pchlide in thylakoids. In contrast to the *flu* mutant, high levels of ^1^O_2_ are produced in response to excess excitation energy in PSII of wild-type plants when photosynthetic activity is inhibited (Mullineaux and Baker, 2010). It is well established that ^1^O_2_ is the major ROS generated under HL conditions (Triantaphylidès *et al.*, 2008; Triantaphylidès and Havaux, 2009) and, consequently, the activation of defence responses under HL conditions ought to share a set of ^1^O_2_-responsive transcripts with the *flu* mutant family. GENEVESTIGATOR can be used to perform comparative genome-wide analyses under different experimental conditions (Hruz *et al.*, 2008), and here it has been used to identify light treatments or *Arabidopsis* mutants that exhibit a transcriptional expression pattern similar to that of ACSC treated with RB. Six experimental conditions are described in GENEVESTIGATOR as HL studies, although the light irradiance used in those studies ranged from 400 μE m^–2^ s^–1^ to 1800 μE m^–2^ s^–1^ (Vandenabeele *et al.*, 2004; Walters *et al.*, 2004; Vanderauwera *et al.*, 2005; Kleine *et al.*, 2007; Rossel *et al.*, 2007; Caldana *et al.*, 2011; González-Pérez *et al.*, 2011). Additionally, the GENEVESTIGATOR database contains the transcriptional profiling of *Arabidopsis* after perturbation with UV radiation, a ‘light’ perturbation also known to lead to the production of ^1^O_2_ and other ROS at high doses (Brosche *et al.*, 2002; Ulm *et al.*, 2004; Hideg *et al.*, 2013). Among the *Arabidopsis* mutants described to produce ^1^O_2_, the database of GENEVESTIGATOR only includes the *flu* mutant family (Laloi *et al.*, 2007), but not others. The CEL files of the double mutant *npq1lut2* were kindly provided by Dr Alboresi and co-workers (Alboresi *et al.*, 2011) and were included in the genome-wide analysis. The study by Ramel and co-workers (2012*b*, 2013) could not be included in the co-regulation analysis because these authors used CATMAv5 instead of Affymetrix microarrays (Ramel *et al.*, 2012*a*, *b*), and only a manual inspection was performed with their microarray data (see the Discussion). Using a list of 400 transcripts of the 0.5 μM RB treatment with |Log_2_|>1, it was found that the biclustering tool of GENEVESTIGATOR identified a group of transcripts exhibiting an expression pattern similar to that described in the *flu* mutant family. Additionally, other treatments such as HL irradiance beyond 1400 μE m^–2^ s^–1^ or UV radiation also proved to have a similar expression pattern. In contrast, HL treatments below 1400 μE m^–2^ s^–1^ showed a poor correlation. In a more robust analysis, the total number of 485 transcripts with significant differential expression (with |Log_2_|>1) in the treatment with 0.5 μM RB was subjected to hierarchical clustering in order to identify both experimental conditions with similar differential transcript expression and clusters of transcripts that could be rich in specific markers for ^1^O_2_.

**Fig. 6.**
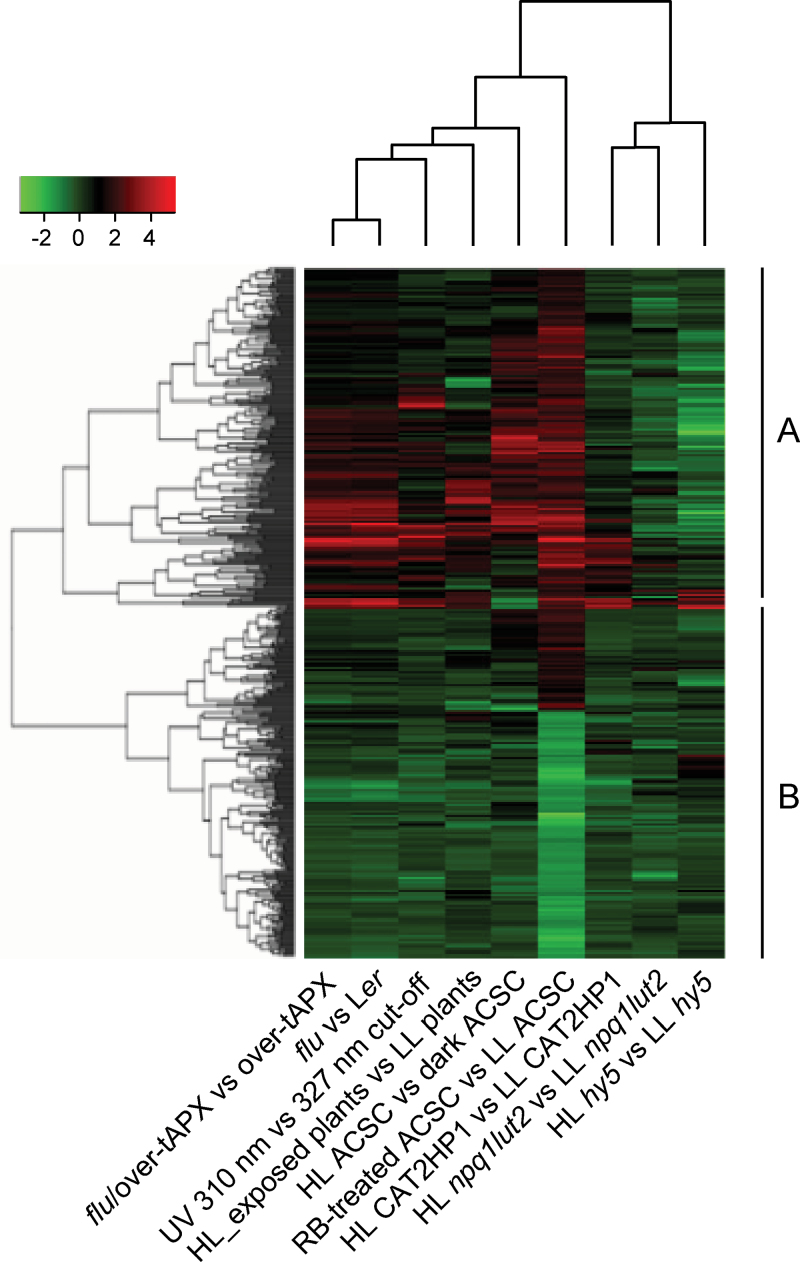
Overall picture of the hierarchical clustering analysis on the 485 transcripts with statistically differential expression and fold change |Log_2_|>1 in ACSC after the treatment with 0.5 μM RB. Data were clustered together with available expression microarray data from HL experiments and *Arabidopsis* mutants included in the GENEVESTIGATOR database: HL *hy5* vs LL *hy5*, *long hypocotyl5* transcription factor mutant of *A. thaliana* (Col-0) seedlings exposed to HL irradiance (1000 μE m^–2^ s^–1^) for 3h versus low light (LL) irradiance (100 μE m^–2^ s^–1^) (Kleine *et al.*, 2009); HL *npq1lut2* vs LL *npq1lut2*, double mutant of 6-week-old plants of *A. thaliana* (Col-0) lacking violaxanthin de-epoxidase and lycopene-ε-cyclase activities exposed to HL irradiance (1000 μE m^–2^ s^–1^) for 2h versus LL irradiance (25 μE m^–2^ s^–1^) (Alboresi *et al.*, 2011); HL CAT2HP1 vs LL CAT2HP1, 6-week-old catalase-deficient plants of *A. thaliana* (Col-0) exposed to HL irradiance (1600–1800 μE m^–2^ s^–1^) for 8h versus LL irradiance (100–140 μE m^–2^ s^–1^) (Vanderauwera *et al.*, 2005); RB-treated ACSC vs LL ACSC, this study; HL ACSC vs dark ACSC, *Arabidopsis* cell suspension cultures exposed to HL irradiance (1000 μE m^–2^ s^–1^) for 0.5h versus 1h dark conditions (González-Pérez *et al.*, 2011); HL-exposed plants vs LL plants; mature wild-type plants of *A. thaliana* (Col-0) exposed to HL irradiance (1400–1600 μE m^–2^ s^–1^) for 1h versus LL irradiance (40–70 μE m^–2^ s^–1^) (Rossel *et al.*, 2007); UV 310nm vs 327nm cut-off; seedlings of *A. thaliana* (Wassilewskija) exposed for 15min at midday to UV-B from Philips TL 40W/12 UV fluorescent tubes unfiltered through a 3mm quartz plate versus filtered through a 3mm transmission WG327cut-off filter (Ulm *et al.*, 2004); *flu* vs L*er*, the *flu* mutant of *A. thaliana* (L*er*) exposed to light irradiance (90 μE m^–2^ s^–1^) for 2h after incubation in the dark for 8h versus dark conditions (Laloi *et al.*, 2007); *flu*/over-tAPX vs over-tAPX, the *flu* mutant of *A. thaliana* (L*er*) overexpressing the thylakoid-bound ascorbate peroxidase (tAPX) exposed to light irradiance (90 μE m^–2^ s^–1^) for 2h after incubation in the dark for 8h versus dark conditions (Laloi *et al.*, 2007). See the text for a detailed description of the main clusters A and B.

In the first instance, it is worth noting that the *hy5* (*long hypocotyl5* transcription factor mutant of *Arabidopsis*) and *npq1lut2* mutants were subjected to light irradiance of 1000 μE m^–2^ s^–1^ (Kleine *et al.*, 2007; Alboresi *et al.*, 2011) and exhibited a poor correlation with the other selected experimental systems (Supplementary Table S9 at *JXB* online); they clustered together with catalase-deficient (CAT2HP1) plants (Fig. 6). CAT2HP1 plants in HL accumulate H_2_O_2_ (Vanderauwera *et al.*, 2005) and exhibit a distinctive transcriptional profile when compared with other experimental systems where ^1^O_2_, ozone (O_3_), or superoxide radical (O_2_^–^) accumulates (Gadjev *et al.*, 2006). The other set of experiments clustered and, in particular, the transcriptional profile of the treatment with 0.5 μM RB showed the highest correlation with the *flu* family mutants (Supplementary Table S9).

In the second instance, two main clusters of transcripts were observed and named A and B (Fig. 6). Cluster A consisted of 242 transcripts and was characterized by the presence of the largest number of specific markers for ^1^O_2_ (i.e. 27 out of 36). The value of 3.42 for the odds ratio [i.e. 27×(242–9)/9×(243–27)] indicated that the enrichment of specific markers for ^1^O_2_ in cluster A was statistically significant (*P*-value of 1.5×10^–3^). Cluster B with 243 transcripts included all the down-regulated transcripts with a fold change <1 in the treatment with 0.5 μM RB and it did not show any significant enrichment in specific markers for any type of ROS. Table 1 summarizes the list of specific markers for ROS identified in cluster A. It is worth noting that six out of the 27 specific markers for ^1^O_2_ identified in cluster A were also found in the list of the 229 up-regulated transcripts with a fold change >1 in the treatment with 500 μM H_2_O_2_ (Supplementary Table S6 at *JXB* online).

**Table 1.** ^1^O_2_ markers with statistically significant enrichment (P<0.05) in cluster A of the hierarchical clustering analysis of the 485 transcripts with fold change |Log_2_|>1 in ACSC after the 30min treatment with 0.5 μM RB

Element	Locus ID	Annotation
248799_at	At5g47230	ERF5, ethylene responsive element binding factor 5
266821_at	At2g44840	ERF13, ethylene responsive element-binding factor 13
248448_at	At5g51190	AP2 (Apetala2) domain-containing transcription factor, putative
262211_at	At1g74930	Member of the dehydration response element-binding (DREB) subfamily A-5 of ERF/AP2 transcription factor family
259992_at	At1g67970	HSFA8, heat shock transcription factor A8
247707_at	At5g59450	SCL11, Scarecrow-like transcription factor 11
252679_at	At3g44260	CCR4-NOT transcription complex protein, putative
257511_at	At1g43000	Zinc-binding family protein
252474_at	At3g46620	Zinc finger (C3HC4-type RING finger) family protein
258436_at	At3g16720	Zinc ion binding/protein binding
256306_at	At1g30370	Lipase class 3 family protein
246600_at	At5g14930	SAG101, senescence-associated gene 101; triacylglycerol lipase
245777_at	At1g73540	NUDT21, Nudix hydrolase homologue 21
248793_at	At5g47240	NUDT8, Nudix hydrolase homologue 8
252592_at	At3g45640	MPK3, mitogen-activated protein (MAP) kinase 3
257751_at	At3g18690	MKS1, MAP kinase substrate 1
255872_at	At2g30360	CIPK11, Cystathionine β-lyase-interacting protein kinase 11
251683_at	At3g57120^*a*^	Protein kinase family protein
252470_at	At3g46930^*a*^	Protein kinase family protein
263320_at	At2g47180	GOLS1, galactinol synthase 1
247811_at	At5g58430	EXO70B1, exocyst subunit EXO70 family protein B1
247240_at	At5g64660^*a*^	U-box domain-containing protein
256999_at	At3g14200^*a*^	DNAJ heat shock N-terminal domain-containing protein
246108_at	At5g28630	Glycine-rich protein
266259_at	At2g27830	Similar to pentatricopeptide (PPR) repeat-containing protein
246270_at	At4g36500^*a*^	Similar to unknown protein (TAIR:At2g18210.1)
245840_at	At1g58420	Similar to unknown protein (TAIR:At1g10140.1)

^*a*^ Specific markers for ^1^O_2_ that are also up-regulated in ACSC after the treatment with 500 μM H_2_O_2_.

## Discussion

### Chloroplast-dependent ^1^O_2_-mediated PCD in ACSC treated with RB

RB, which accumulates in chloroplasts of *Arabidopsis* cells, caused a surge in ^1^O_2_ production that activated transcriptional defence responses leading to the physiological induction of PCD. However, treatment with MV or IC, which do not enter chloroplasts, did not activate any statistical change in the transcriptional profile of ACSC. In spite of this, it could not be unambiguously concluded that the divergent subcellular location of the three ^1^O_2_ elicitors was the sole cause of this observation because the ^1^O_2_ quantum yields of the three elicitors are inherently different and, in particular, lower for MV and IC (Kovacs *et al*., 2013). Therefore, this physiological defence response was investigated in more detail in both dark-grown and light-grown *Arabidopsis* cells in order to establish whether its activation was initiated by ^1^O_2_ production inside the chloroplasts or instead was independent of cellular location. It was determined that PCD was not activated by ^1^O_2_ produced by RB in dark-grown cells that had not developed chloroplasts; a result that was in full agreement with the recent study by Kim and co-workers (2012), who observed that the ^1^O_2_-mediated cell death in seedlings of the leaf-variegated mutant *variegated2* was only induced in cells of the green leaf sector of this mutant containing fully developed chloroplasts, but not in the white sector where undifferentiated plastids were present. Chloroplasts are known both to be an important source of defence signalling molecules (i.e. ROS and precursors of defence hormones) and to have a central role in defence responses and the hypersensitive response (HR), a form of PCD localized at the site of pathogen attack that requires light for its development in many cases (Karpinski *et al.*, 2003; Coll *et al.*, 2011; Kacprzyk *et al.*, 2011). Likewise, the involvement of chloroplasts and ROS in PCD was recently demonstrated in ACSC (Doyle *et al.*, 2010), supporting the view that chloroplasts play a significant role in PCD regulation. In some studies carried out with *C. reinhardtii*, it has been proposed that ^1^O_2_ can escape from the chloroplasts and interact with an ^1^O_2_ sensor located in the cytoplasm or even reach the nucleus (Fischer *et al.*, 2007). However, on the basis of the present results with RB, which does not activate PCD in dark-grown cells, it is difficult to reconcile the results with a role for ^1^O_2_ itself as a signalling molecule outside chloroplasts unless extremely, non-physiological HL conditions or very high concentrations of ^1^O_2_ elicitors come into play. Hence it is proposed that ^1^O_2_-mediated PCD in ACSC is initiated only via developed chloroplasts.

Under the experimental conditions used here, 500 μM H_2_O_2_ did not activate PCD in cells grown in dark or light conditions. Low PCD rates in ACSC subjected to H_2_O_2_ were shown here; however, higher H_2_O_2_ concentrations or longer incubation times than those described in this study were required to increase the PCD levels (Desikan *et al*., 1998, 2001).

The functional enrichment analysis of the transcriptional profiling of light-grown ACSC also provided lines of evidence for the activation of PCD after the treatment with 0.5 μM RB, but not with 500 μM H_2_O_2_. A direct comparison between the 30min, 2-fold up-regulated transcripts in ACSC treated with 0.5 μM RB and the 30min early-induced, 2-fold up-regulated transcripts in the *flu* mutant (op den Camp *et al.*, 2003) showed that >40% of the up-regulated transcripts in ACSC (i.e. 138 of out 314) were included in the above list of up-regulated transcripts in the *flu* mutant. Likewise, the list of the 30min, 2-fold, up-regulated transcripts in ACSC, treated with 0.5 μM RB, contained 32 specific markers for ^1^O_2_ (Supplementary Table S6 at *JXB* online), seven specific markers for H_2_O_2_, and no specific markers for O_2_^–^ when it was compared with lists of specific markers for ROS provided by Gadjev and co-workers (2006). This indicated that ^1^O_2_ produced by RB in ACSC induced the expression of a list of transcripts that resembled the list observed in the *flu* mutant and confirmed that the transcriptional defence responses observed were mediated by ^1^O_2_ production inside chloroplasts. However, they did not seem to be associated with the EX1/EX2- and EDS1-dependent signalling pathway (see below). In addition, the analysis of the over-represented GO/biological process terms in *Arabidopsis* cells treated with 0.5 μM RB showed that most of them corresponded to responses to pathogens, several biotic and abiotic stimuli, and PCD, which were also observed in the *flu* mutant (op den Camp *et al.*, 2003). Unexpectedly, the list of the 2-fold up-regulated transcripts in ACSC after the treatment with 500 μM H_2_O_2_ also exhibited some overlap (<30%) with the list of the 30min early-induced, 2-fold up-regulated transcripts in the *flu* mutant and 19 specific markers for ^1^O_2_ (Supplementary Table S6), and only three specific markers for H_2_O_2_ were identified. The fact that the treatment with 500 μM H_2_O_2_ inhibited the expression of several chloroplast transcripts (Log_2_ <1) particularly associated with photosynthesis (Supplementary Table S3, locus identifier in a cyan background) suggested that H_2_O_2_ clearly entered chloroplasts and activated transcriptional defence responses that had common characteristics with those described for the 0.5 μM RB treatment and the *flu* mutant. This is confirmed in Supplementary Tables S7 and S8, where a significant number of over-represented GO/biological process terms after the treatments with 0.5 μM RB and 500 μM H_2_O_2_ appeared in both lists. However, a closer inspection of both lists disclosed that GO terms such as resistance response to pathogen (GO:0009814 and GO:009627), protein ubiquitination (GO:0016567), and PCD (GO:0012501 and GO:0006915) were only present in the treatment with 0.5 μM RB. The list of the 30min, up-regulated transcripts in ACSC after the treatment with 0.5 μM RB contained key transcripts associated with the above GO terms that included: resistance (*R*) transcripts encoding TIR-NB-LRR-type resistance and CC-NB-LRR proteins (At5g41750; At5g41740; At1g58807; At1g59124; At1g59620), MITOGEN-ACTIVATED PROTEIN KINASES (*MPK3*, At3g45640; *MPKK4*, At1g51660), the ubiquitin ligase PLANT U-BOX17 (*PUB17*, At1g29340), a NUDIX HYDROLASE HOMOLOG (*NUDT7*, At4g12720), BCL-2-ASSOCIATED ATHANOGENE (*BAG3*, At5g07220), the lipase-like protein PHYTOALEXIN DEFICIENT 4 (*PAD4*, At3g52430), the ELICITOR PEPTIDE 2 PRECURSOR (*PROPEP2*, At5g64890), the transcription factor WRKY70 (At3g56400), and the ANKYRIN REPEAT-CONTAINING PROTEIN 2A (*AKR2A*; At4g35450); the underlined transcripts of which were also present in the early-induced, 2-fold up-regulated transcripts of the *flu* mutant. In contrast, the list of up-regulated transcripts in ACSC treated with 500 μM H_2_O_2_ contained only four transcripts associated with PCD and resistance response to pathogens (*BAG3*, At5g07220; *PROPEP2*, At5g64890; *AKR2A*, At4g35450; and *MYB30*, At3g28910), but none of them was included in the list of the early-induced, 2-fold up-regulated transcripts in the *flu* mutant.

It has been proposed that ethylene (ET) and salicylic acid (SA) signalling pathways function additionally during the ^1^O_2_-mediated PCD and that the oxylipins 12-oxophytodienoic acid (OPDA) and dinor-OPDA antagonize the cell death-inducing activity of jasmonic acid (JA) in the *flu* mutant (Danon *et al.*, 2005). In a search for key transcripts associated with the biosynthesis of the above enzymes in ACSC treated with 0.5 μM RB, it was observed that 1-AMINOCYCLOPROPANE-1-CARBOXYLIC ACID (ACC) SYNTHASE 6 (*ACC6*, At4g11280) was present together with another eight ethylene-responsive element binding factors (ERFs) that included all those early-induced ERFs listed by Danon and co-workers (2005). In addition, transcripts encoding enzymes associated with the biosynthesis of OPDA in chloroplasts such as ALLENE OXIDE SYNTHASE (*AOS*, At5g42650), the conjugation of OPDA with glutathione such as GLUTATHIONE *S*-TRANSFERASE 5 (*GST5*, At2g29450), and the biosynthesis of JA in peroxisomes such as 3-oxo-2-[2’(Z)-pentenyl]-cyclopentane-1-octanoic (OPC-8:0) CoA LIGASE 1 (*OPCL1*, At1g20510) were also present. However, there was no evidence for the up-regulation of *EDS1* (At3g48090) known to be required for the biosynthetic activation of SA and the modulation of the ^1^O_2_-mediated PCD in the *flu* mutant (Ochsenbein *et al.*, 2006). The essential regulation role of EDS1 is mediated by its interaction with PAD4, and also by the product of the *SENESCENCE-ASSOCIATED GENE 101* (*SAG101*, At5g14930), two proteins that have defence regulatory functions beyond stabilizing EDS1 in TIR-NB-LRR-type *R* gene-triggered resistance as demonstrated by silencing *EDS1* with a double-standed DNA (dsRNA) interference construct (Feys *et al.*, 2005). The evidence that key transcripts associated with the biosynthesis pathway of ET and JA, together with *PAD4* and *SAG101*, are up-regulated in ACSC with 0.5 μM RB suggests that they could be responsible for the induction of PCD through a signalling pathway that does not depend on EDS1. This agrees with the recent study by Ramel *et al.* (2013), who established that ^1^O_2_ activated cell death in the *ch1* mutant under HL stress, although no evidence for the activation of the EX1/EX2- and EDS1-dependent signalling pathway was found. In contrast, JA was described to have a key role in the ^1^O_2_-mediated cell death, finding significant differences in the activation or repression of transcripts associated with the biosynthesis of this hormone under HL or acclimatory conditions. It is worth noting that an acclimatory response, but not PCD, was activated in ACSC subjected to a 30min treatment with HL (1800 μE m^−2^ s^−1^) (González-Pérez *et al.*, 2011; Gutiérrez *et al.*, 2011) and that no evidence was found for the up-regulation of *EDS1*, *PAD4* and *SAG101* nor for transcripts encoding enzymes associated with the biosynthesis of JA in peroxisomes.

Similarly, a search for key early-induced transcripts associated with the biosynthesis and signalling cascade of ET, JA, and SA (listed in the study by Danon and co-workers, 2005) in ACSC treated with 500 μM H_2_O_2_ did not yield any match except for a transcript encoding the transcription factor ERF6 (At4g17490), supporting the view that PCD was not activated either. Of interest is the observation that ACSC treated with 500 μM H_2_O_2_ exhibited several up-regulated transcripts encoding UDP-glycosyl transferases (UGTs) and GST, some of them known to be early-induced by H_2_O_2_, such as *UGT73B3*, *UGT73B5*, and *GST6* and with an important role in plant defence responses (Chen and Singh, 1999; Langlois-Meurinne *et al.*, 2005).

### Correlation between the *flu* family mutants and ACSC treated with RB

The hierarchical clustering analysis of 485 co-regulated transcripts between ACSC treated with 0.5 μM RB, ^1^O_2_-producing mutants, and wild-type plants subjected to HL showed that ACSC treated with 0.5 μM RB had higher Pearson’s correlation with the *flu* family mutants than with the *npq1lut2* mutant. ACSC treated with 0.5 μM RB had 27 specific markers for ^1^O_2_ in common with the *flu* mutant (Table 1), but only two out of the 27 were up-regulated in the *npq1lut2* mutant exposed to HL for 2h (At5g47240 and At3g14200), where an acclimatory response was reported (Alboresi *et al.*, 2011). A manual inspection of the up-regulated transcripts (with Log_2_ ratios ≥1) in *ch1* (Ramel *et al.*, 2013) showed that four (At2g29450, At2g30360, At2g44840, and At3g44260) were listed among the 27 specific markers for ^1^O_2_ in cluster A when this mutant was subjected to HL for 2 d, but none when *ch1* was first acclimated to HL. Interestingly, the former up-regulated transcripts are involved in response to ET and JA stimuli. A transcriptional analysis of defence responses mediated by carotenoid oxidation products (i.e. β-cyclocitral) with origin in the reaction between ^1^O_2_ and the β-carotene molecules (housed in the PSII RC) also proved that ^1^O_2_ production by PSII RC induced an acclimatory rather than a PCD response (Ramel *et al.*, 2012*a*, *b*). A manual inspection of the up-regulated transcripts in the aforementioned study also indicated that only two out of the 27 specific markers for ^1^O_2_ in cluster A (At3g46620 and At3g14200) were up-regulated after the treatment with β-cyclocitral. In the study by Rossel and co-workers (2007), leaves were directly exposed to HL for 30min. The list of up-regulated transcripts contained several specific markers for ^1^O_2_, eight of which were included in cluster A (At1g74930, At2g30360, At2g35710, At2g44840, At2g47180, At3g44260, At3g46930, At5g47240, see Table 1 for their description). Despite the fact that this HL treatment induced a significant number of specific markers for ^1^O_2_, the underlined transcripts of which were involved in response to ET and JA stimuli, the authors described the activation of a (systemic acquired) acclimatory response. The production of ^1^O_2_ and other ROS has also been demonstrated in plants exposed to high doses of UV, and their effects on the expression of transcripts encoding enzymes with an antioxidant role and with a UV-dependent expression have been recently investigated (Hideg *et al.*, 2013). However, the overlap between transcripts encoding several antioxidative enzymes in the *flu* mutant and the UV-induced transcripts was very limited. In fact, a single match with the transcript encoding GLUTAREDOXIN (*GRX480*, At1g28480)—also induced in other chemical treatments—was detected. A similar conclusion was reached when the 14 UV-induced transcripts were compared with the 2-fold, up-regulated transcripts in ACSC treated with 0.5 μM RB, as only *GRX480* was also up-regulated. Although the hierarchical clustering analysis shows certain correlation between experiments where ^1^O_2_ is produced, the genome-wide expression profiling and, consequently, the ^1^O_2_-mediated defence responses (i.e. acclimation or PCD) vary significantly between experimental conditions, where the EX1/EX2- and EDS1-dependent signalling pathway is not activated or overlaps with other ^1^O_2_-mediated signalling pathways belonging to a more complex signalling network.

### Conclusion

In brief, PCD has been activated in *Arabidopsis* cell cultures treated with submicromolar concentrations of RB, a potent, artificial ^1^O_2_ photosensitizer that accumulates inside chloroplasts. The fact that this ^1^O_2_-mediated physiological response occurs in light-grown cultures, but not in dark-grown cultures, suggests that its activation requires functional chloroplasts. Additionally, a co-regulation analysis using the early-induced transcripts with a statistically significant fold change |Log_2_|>1 in ACSC treated with RB showed that this treatment had high correlation with the *flu* family mutants (although there was no evidence for the up-regulation of *EDS1*), but low correlation with other ^1^O_2_-producing mutants or HL treatments in which an acclimatory response was activated instead of cell death.

## Supplementary data

Supplementary data are available at *JXB* online.

Figure S1. Volcano plots of the microarray experiments:

Figure S2. Validation of the microarray experiments in ACSC after the 30min treatments with 0.5 μM RB and 500 μM H_2_O_2_.

Table S1. Transcripts and their corresponding primers selected for monitoring early ROS-mediated responses in ACSC under (photo)oxidative stress.

Table S2. Set of transcripts down- and up-regulated in ACSC after the 30min treatment with 0.5 μM RB (adjusted *P*-value <0.05).

Table S3. Set of transcripts down- and up-regulated in ACSC after the 30min treatment with 500 μM H_2_O_2_ (adjusted *P*-value <0.05).

Table S4. Transcripts and their corresponding primers selected for validation of the microarray experiments in ACSC under (photo)oxidative stress.

Table S5. Fold changes (Log_2_) in the expression of selected transcripts responding to ROS production in ACSC after the 30min treatment with RB, MV, and IC at 0.5 μM, and H_2_O_2_ at 500 μM.

Table S6. Up-regulated specific markers for ^1^O_2_ in ACSC after several chemical treatments.

Table S7. Tree view of over-represented GO/biological process terms in *Arabidopsis* cell suspension culture after the 30min treatment with 0.5 μM RB (adjusted *P*-value <0.05).

Table S8. Tree view of the over-represented GO/biological process terms in *Arabidopsis* cell suspension culture after the 30min treatment with 500 μM H_2_O_2_ (adjusted *P*-value <0.05).

Table S9. Pearson’s correlation between different experimental conditions and *Arabidopsis* mutants.

Supplementary Data

## Abbreviations:

ACSC: *Arabidopsis thaliana* cell suspension cultures
Chl: chlorophyll
DAB: 3,3′-diaminobenzidine tetrahydrochloride
DNPH: 2,4-dinitrophenylhydrazine
ET: ethylene
EX: EXECUTER
FDA: fluorescein diacetate
HL: high light
IC: indigo carmine
JA: jasmonic acid
LL: low light
MV: Methylene Violet
OPDA: 12-oxophytodienoic acid
PCD: programmed cell death
Pchlide: protochlorophyllide
RB: Rose Bengal
ROS: reactive oxygen species
SA: salicylic acid.
