# Supplementary material for: Programmed cell death activated by Rose Bengal in Arabidopsis thaliana cell suspension cultures requires functional chloroplasts
Source: J Exp Bot. 2014 Apr 10;65(12):3081–95. doi: 10.1093/jxb/eru151 (PMC4071827; doi:10.1093/jxb/eru151)
Supplement: Supplementary Data [file supp_65_12_3081__index.html]

Programmed cell death activated by Rose Bengal in Arabidopsis thaliana cell suspension cultures requires functional chloroplasts — Programmed cell death activated by Rose Bengal in Arabidopsis thaliana cell suspension cultures requires functional chloroplasts — Supplementary Data 

# Programmed cell death activated by Rose Bengal in *Arabidopsis thaliana* cell suspension cultures requires functional chloroplasts

## Supplementary Data

Data files

**Files in this Data Supplement:**

- Supplementary Data - Supplementary Data
- Supplementary Data - Supplementary Data
- Supplementary Data - Supplementary Data
