# Supplementary material for: Programmed cell death activated by Rose Bengal in Arabidopsis thaliana cell suspension cultures requires functional chloroplasts
Source: J Exp Bot. 2014 Apr 10;65(12):3081–95. doi: 10.1093/jxb/eru151 (PMC4071827; doi:10.1093/jxb/eru151)
Supplement: Supplementary Data [file supp_eru151_jexbot120030_file001.pdf]

Figure S1

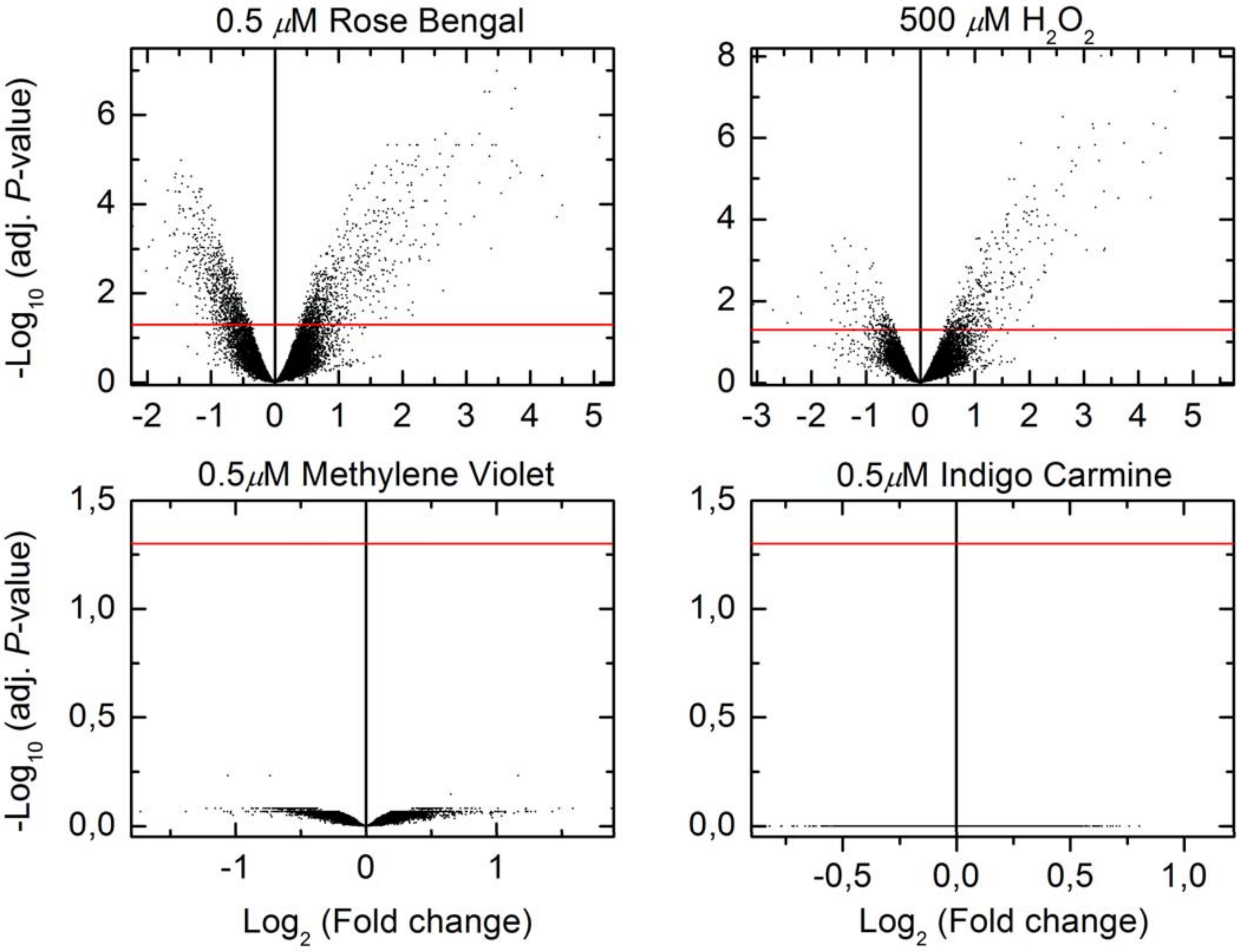

Supplemental Figure S1. Volcano plots of the microarray experiments: For each transcript in every comparative treatment the negative Log_{10} of the adjusted *P*-value is represented versus Log_2 of the fold change. Whereas the treatments with 0.5 μM RB and 500 μM H_2O_2 clearly show transcripts with statistically significant differential expression (dots above the horizontal red line, adjusted p-value < 0.05), the treatments with 0.5 μM IC and 0.5 μM MV did not exhibit any transcripts with statistically significant differential expression (all dots are below horizontal red line).

Figure S2

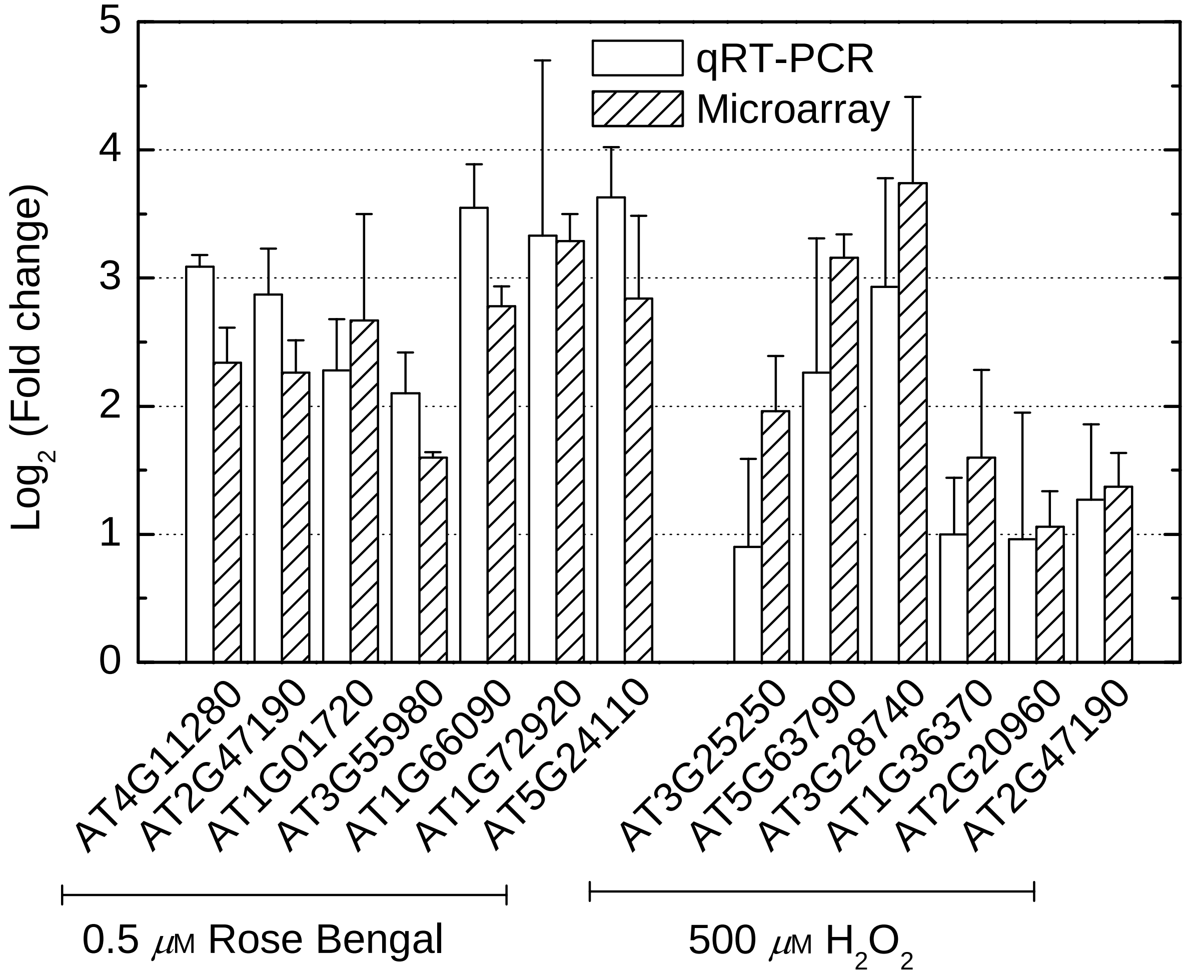

Supplemental Figure S2. Validation of the microarray experiments in ACSC after the 30-min treatments with 0.5 µM RB and 500 µM H_2O_2. Selected transcripts showed statistically significant differential expression under the assayed experimental conditions (adjusted *p*-value < 0.05).

Table S1. Transcripts and their corresponding primers selected for monitoring early ROS-mediated responses in ACSC under (photo)oxidative stress.

**Locus Identifier**	**Name**	**Marker**	**Expression**[a]	**Primers**
At2g43510	*DEF, DEFENSIN-LIKE FAMILY PROTEIN*	Oxidative stress	Response to fungus, herbivores and general abiotic stress	F: 5´- GGCTATCGTTTCCATCTTCGT -3´<br>R: 5´- CCAACATCACCTCCGTATTCTT -3´
At5g64870	*NOD, NODULIN-LIKE PROTEIN*	$^1O_2$	Up-regulated in the presence of $^1O_2$	F: 5´- GAGGTCTTCAAAGGAACAAAGGAG -3´<br>R: 5´- CTGCTTCACATTGGCGTTG -3´
At1g17380	*JAZ5, JASMONATE-ZIM-DOMAIN PROTEIN 5*	$^1O_2$	Response to jasmonic acid stimulus and HL stress	F: 5´- ATTCCAGTCTCGCGTTGC -3´<br>R: 5´- AGGTAGAGGGTTCGCCTTTG -3´
At3g55980	*SZF1, SALT-INDUCIBLE ZINC FINGER PROTEIN 1*	$^1O_2$	Response to MeJA, chitin, salt, pathogens and HL stress	F: 5´- ACCCACCACAACACAATTCC -3´<br>R: 5´- TTGCTCTTTGGTCCACTGC -3´
At4g21650	*SUBT, SUBTILASE FAMILY PROTEIN*	H_2O_2	Involved in proteolysis	F: 5´- TGGAGCCTTAGACAGTGATAGC -3´<br>R: 5´- GATTCAAGCATCTGGTGATGAG -3´
At5g26280	*F9D12, TRAF-LIKE FAMILY PROTEIN*	H_2O_2	Not described	F: 5´- TAGGTCACAAAGGGCAGAGG -3´<br>R: 5´- GATGGTCGCTCTTCTCTCAAC -3´
At4g05040	*C17L7, ANKYRIN REPEAT FAMILY PROTEIN*	H_2O_2	Not described	F: 5´- GATAACGGCTTAACAGCTAGGG -3´<br>R: 5´- GCAGCAAACGTCATTGTGG -3´
At2g19760	*PROF, PROFILIN FAMILY PROTEIN*	Internal Control	Constitutive gene	F: 5´- CCAAGGTGAACAAGGAGCTG -3´<br>R: 5´- CAAGTTGCATTGACCTCCAG -3´

[a]Gene expression in response to several environmental/biological conditions: *DEF* (Gadjev et al. 2006); *NOD*, *JAZ5*, *SZF1*, *SUBT*, *F9D12*, *C17L7* (Op den Camp et al., 2003; Gadjev et al., 2006; González-Pérez et al., 2011), and *PROF* (Laloi et al., 2007).

Table S4. Transcripts and their corresponding primers selected for validation of the microarray experiments in ACSC under (photo)oxidative stress.

Treatment	Locus Identifier	Annotation	Primers
0.5 μM RB	At5g63790	ANAC, NAC domain containing protein 102; transcription factor	F: 5´- CGGAGATGTGCGTCAGAAC-3´<br>R: 5´- TCCGGTCTCTATGCGAGAAG-3´
	At4g11280	ACC, 1-aminocyclopropane-1-carboxylic acid synthase 6	F: 5´- CGGCGATGGTTTCTTAGTTC-3´<br>R: 5´- AGGCTTCCACCGTAATCTTG-3´
	At1g72920	TIR-NBS, toll-interleukin-resistance domain family protein	F: 5´- GAGACGAGCATTGACCAGTTTAG -3´<br>R: 5´- CCCTCCCATTGCTTATTGTTG -3´
	At1g01720	ATAF1, NAC domain containing protein 2; transcription factor	F: 5´- GCAAAGCTCCAAAGGGAGAG -3´<br>R: 5´- TGTAAATCCGGCAGAGAACC -3´
	At3g55980	SZF1, zinc finger (CCCH-type) family protein	F: 5´- ACCCACCACAACACAATTCC-3´<br>R: 5´- TTGCTCTTTGGTCCACTGC-3´
	At1g66090	TIR-NBS, toll-interleukin-resistance domain family protein	F: 5´- CCACGCTTTGACCTGTGTG-3´<br>R: 5´- GATCATGTTTGCCTCATCGTC-3´
	At5g24110	WRKY30, WRKY DNA-binding protein 30; transcription factor	F: 5´- TGGACGATGGATTCAGTTGG -3´<br>R: 5´- TGTTTAGTGGCTTCACATCCTTG -3´
500 μM H_2O_2	At1g36370	SHM7, serine hydroxymethyltransferase 7	F: 5´- ATGCAGACTGGTTACTGGTGG -3´<br>R: 5´- ATGGCACATCTCACACACTTTC -3´
	At3g28740	CYP81, cytochrome P450 family protein	F: 5´- GCCTGATTACTACACGGATGTGA -3´<br>R: 5´- GATGTATCAGTCCCGGCAAG -3´
	At2g47190	MYB2, myb domain protein 2; transcription factor	F: 5´- CACATCGCTCGTTCCTCTG -3´<br>R: 5´- GTTGCCTCTTCTAACATCTGGAC -3´
	At2g20960	PEARL4, phospholipase-like protein 4	F: 5´- GGCAATCCTTTCCATGAGTG -3´<br>R: 5´- GAGGAGTCTGATCCCTGCTG -3´
	At3g25250	OXI1, oxidative signal-inducible1; kinase	F: 5´- AGGAAAATGCAATCGGAAAG-3´<br>R: 5´- TTGGATCATCACATTGTCTGG-3´
	At5g63790	ANAC, NAC domain containing protein 102; transcription factor	F: 5´- CGGAGATGTGCGTCAGAAC-3´<br>R: 5´- TCCGGTCTCTATGCGAGAAG-3´

Table S5. Fold changes (Log_2) in the expression of selected transcripts responding to ROS production in ACSC after the 30-min treatment with RB, MV and IC at 0.5 μM, and H_2O_2 at 500 μM. * p-value < 0.05

**Transcript**	**Marker**	**RB**		**MV**		**IC**		**H_2O_2**	
		Light	Dark	Light	Dark	Light	Dark	Light	Dark
NOD	$^1O_2$	1.32± 0.33*	0.42± 0.58	0.20± 0.76	-0.24± 0.29	0.36± 0.35	-0.54± 0.02	-0.01± 0.12	0.02± 0.41
JAZ5	$^1O_2$	-0.06± 0.67	0.35± 0.66	-0.22± 0.69	-0.06± 0.50	0.57± 0.69	0.06± 0.16	0.26± 1.18	0.91± 1.16
SZF1	$^1O_2$	1.48± 0.73*	0.31± 0.21	0.44± 0.59	0.03± 0.37	0.24± 0.24	-0.39± 0.34	0.69± 0.62	0.76± 0.91
SUBT	H_2O_2	-2.80± 0.98*	-0.20± 0.90	-0.07± 0.27	-0.24± 0.43	0.03± 0.26	-0.47± 0.60	-0.45± 0.51	-0.12± 1.11
F9D12	H_2O_2	-0.99± 0.61	-0.07± 0.23	0.16± 0.34	0.08± 0.29	0.10± 0.26	-0.38± 0.08	-0.08± 0.60	-0.04± 0.47
C17L7	H_2O_2	-0.30± 1.14	0.03± 0.94	0.16± 0.12	-0.03± 0.58	-0.51± 0.67	0.08± 0.57	0.23± 0.86	-0.21± 0.71
DEF	Oxidative stress	-0.05± 0.85	0.09± 0.40	0.31± 0.18	-0.04± 0.15	0.14± 0.29	0.17± 0.39	0.67± 0.52	0.76± 0.26*

Table S6. Up-regulated specific markers for $^1O_2$ in ACSC after several chemical treatments.

ACSC treated with 0.5 μM RB

ACSC treated with 500 μM H_2O_2

Locus Identifier	*Description*
At5g47240	NUDT8, *Arabidopsis thaliana* Nudix hydrolase homolog 8
At1g58420	Similar to unknown protein (TAIR:AT1G10140.1)
At3g16720	ATL2; Protein binding / zinc ion binding
At2g27830	Similar to pentatricopeptide (PPR) repeat-containing protein
At1g43000	Zinc-binding family protein
At1g30370	Lipase class 3 family protein
At5g59450	SCL11, Scarecrow-like transcription factor 11
At3g45640	MPK3, Mitogen-Activated Protein Kinase 3
At5g47230	ERF5, Ethylene Responsive Element Binding Factor 5
At5g14930	SAG101, Senescence-Associated Gene 101; triacylglycerol lipase
At3g44260	CCR4-NOT, Transcription complex protein, putative
At5g51190	AP2 domain-containing transcription factor, putative
At1g73540	NUDT21, *Arabidopsis thaliana* Nudix hydrolase homolog 21
At2g30360	CIPK11, SOS3-interacting protein 4
At1g74930	ORA47; DNA binding / transcription factor
At5g58430	EXO70B1, Exocyst subunit EXO70 family protein B1
At1g67970	HSFA8, Heat shock transcription factor A8
At3g18690	MKS1, MAP Kinase Substrate 1
At2g44840	ERF13, Ethylene-Responsive Element Binding Factor 13
At5g28630	Glycine-rich protein
At2g47180	GOLS1, Galactinol Synthase 1
At3g06490	MYB108, MYB Domain Protein 108
At2g35710	Glycogenin glucosyltransferase
At5g42650	AOS, Allene Oxide Synthase
At5g64660	U-box domain-containing protein
At3g57120	Protein kinase family protein
At3g46930	Protein kinase family protein
At4g36500	Similar to unknown protein (TAIR:AT2G18210.1)
At3g14200	DNAJ heat shock N-terminal domain-containing protein
At3g46620	Zinc finger (C3HC4-type RING finger) family protein
At2g18210	similar to unknown protein (TAIR:AT4G36500.1)
At2g29450	GSTU5, Glutathione S-transferase (class tau) 5
At4g11350	Transferase, transferring glycosyl groups
At3g25730	AP2 domain-containing transcription factor, putative
At1g14040	ATPase, coupled to transmembrane movement of substances
At5g10830	Embryo-abundant protein-related
At1g51620	Protein kinase family protein
At4g28350	Lectin protein kinase family protein
At1g11670	MATE efflux family protein
At5g56980	Similar to unknown protein (TAIR:AT4G26130.1)
At5g16970	AER, 2-Alkenal reductase
At5g44070	CAD1,Cadmium Sensitive 1
At1g09940	HEMA2; Glutamyl-tRNA reductase

Table S7. Tree view of over-represented GO/biological process terms in Arabidopsis cell suspension culture after the 30-min treatment with 0.5 μM RB (adjusted p-value < 0.05).

GO accession	*Synonyms*
	**Response to stimulus**
GO:0042221[a]	Response to chemical stimulus
GO:0010033	Response to organic substance
GO:0009743	Response to carbohydrate stimulus
GO:0010200[a]	Response to chitin
GO:0009751[a]	Response to salicylic acid stimulus
GO:0009863	Salicylic acid mediated signalling pathway
GO:0009636[a]	Response to toxin
GO:0006979[a]	Response to oxidative stress
GO:0006955[a]	Immune response
GO:0045087[a]	Innate immune response
GO:0009814	Resistance response to pathogen
GO:0009627	Salicylic acid-dependent systemic resistance
GO:0006950[a]	Response to stress
GO:0006952[a]	Defence response
GO:0031347	Down-regulation of defence response.
GO:0031348[a]	Negative regulation of defence response
GO:0009611	Response to wounding
GO:0006970	Response to osmotic stress
GO:0009628[a]	Response to abiotic stimulus
GO:0009266[a]	Response to temperature stimulus
GO:0009408[a]	Response to heat
GO:0009409	Response to cold
GO:0009607[a]	Response to biotic stimulus
GO:0051707[a]	Response to other organism
GO:0009617[a]	Response to bacterium
	**Metabolic process**
GO:0051171	Regulation of nitrogen compound metabolic process
GO:0006139	Nucleobase, nucleoside, nucleotide and nucleic acid metabolic process
GO:0006350	Transcription
GO:0045449	Regulation of transcription
GO:0010468	Regulation of gene expression
GO:0016070	RNA metabolic process
GO:0044267[a]	Cellular protein metabolic process
GO:0006457[a]	Protein folding
GO:0043687[a]	Post-translational protein modification
GO:0016567	Protein ubiquitination
GO:0051603	Proteolysis involved in cellular protein catabolic process
GO:0019941	Modification-dependent protein catabolic process
GO:0006520[a]	Cellular amino acid metabolic process
	**Cellular process**
GO:0007049[a]	Cell cycle
GO:0007017[a]	Microtubule-based process
	**Developmental process**
GO:0012501	Programmed cell death
GO:0006915	Apoptosis

[a]GO terms also over-represented in the treatment with 500 μM H_2O_2

Table S8. Tree view of the over-represented GO/biological process terms in Arabidopsis cell suspension culture after the 30-min treatment with 500 μM H_2O_2 (adjusted *p*-value < 0.05).

GO accession	*Synonyms*
	**Response to stimulus**
GO:0042221[a]	Response to chemical stimulus
GO:0006979[a]	Response to oxidative stress
GO:0009751[a]	Response to salicylic acid stimulus
GO:0009636[a]	Response to toxin
GO:0009407	Toxic catabolic process
GO:0010200[a]	Response to chitin
GO:0010038	Response to metal ion
GO:0046686	Response to cadmium ion
GO:0006955[a]	Immune response
GO:0045087[a]	Innate immune response
GO:0006950[a]	Response to stress
GO:0006952[a]	Defence response
GO:0031348[a]	Negative regulation of defence response
GO:0009628[a]	Response to abiotic stimulus
GO:0009266[a]	Response to temperature stimulus
GO:0009408[a]	Response to heat
GO:0009607[a]	Response to biotic stimulus
GO:0051707[a]	Response to other organism
GO:0009617[a]	Response to bacterium
GO:0009605	Response to external stimulus
	**Metabolic process**
GO:0007154	Cell communication
GO:0007165	Signal transduction
GO:0007242	Intracellular signalling cascade
GO:0010017	Red or far red light signalling pathway
GO:0006793	Phosphorus metabolic process
GO:0006796	Phosphate metabolic process
GO:0016311	Dephosphorylation
GO:0006468	Protein amino acid phosphorylation
GO:0006470	Protein amino acid dephosphorylation
GO:0044267[a]	Cellular protein metabolic process
GO:0006457[a]	Protein folding
GO:0006464	Protein modification process
GO:0043687[a]	Post-translational protein modification
GO:0019752	Carboxylic acid metabolic process
GO:0009308	Amine metabolic process
GO:0006520[a]	Cellular amino acid metabolic process
GO:0006396	RNA processing
GO:0006364	rRNA processing
GO:0019748	Secondary metabolism
GO:0046907	Intracellular transport
	**Cellular process**
GO:0007049[a]	Cell cycle
GO:0051301	Cell division
GO:0007017[a]	Microtubule-based process
GO:0007018	Microtubule-based movement
GO:0051276	Chromosome organization
	**Developmental process**
GO:0008219	Cell death

[a]GO terms also over-represented in the treatment with 0.5 μM RB

Table S9. Pearson's correlation between different experimental conditions and arabidopsis mutants[a].

	flu/over-tAPX vs over-tAPX	*flu* vs Columbia	UV 310 nm vs 327 nm	HL exposed vs LL plants	HL ACSC vs dark ACSC	RB-treated ACSC vs LL	HL CAT2HP1 vs LL CAT2HP1	HL *npq1lut2* vs LL *npq1lut2*	HL *hy5* vs LL *hy5*
flu/over-tAPX vs over-tAPX	1.0000	0.9741	0.6782	0.6520	0.5276	0.6894	0.6350	0.2374	-0.1945
flu vs Ler		1.0000	0.6955	0.6652	0.5075	0.6960	0.6142	0.2412	-0.1683
UV 310 nm vs 327 nm cut-off			1.0000	0.4014	0.4203	0.6195	0.5434	0.0966	-0.2173
HL exposed plants vs LL				1.0000	0.3600	0.4529	0.3735	0.1611	-0.1047
HL ACSC vs dark ACSC					1.0000	0.5939	0.0818	-0.0253	-0.5713
RB-treated ACSC vs LL						1.0000	0.4479	0.2354	-0.2852
HL CAT2HP1 vs LL CAT2HP1							1.0000	0.2457	0.1376
HL *npq1lut2* vs LL *npq1lut2*								1.0000	0.2164
HL *hy5* vs LL *hy5*									1.0000

[a]Microarray data for the selected experimental conditions and arabidopsis mutants were obtained from the following references: op den Camp et al., 2003; Gadjev et al., 2006; Brosche et al., 2002; Ulm et al., 2004; Rossel et al., 2007; González-Pérez et al., 2011; this study; Vanderauwera et al., 2005; Alboresi et al., 2011; Kleine et al., 2007.
